# Safety and efficacy of anti-PCSK9 antibodies: a meta-analysis of 25 randomized, controlled trials

**DOI:** 10.1186/s12916-015-0358-8

**Published:** 2015-06-23

**Authors:** Xin-Lin Zhang, Qing-Qing Zhu, Li Zhu, Jian-Zhou Chen, Qin-Hua Chen, Guan-Nan Li, Jun Xie, Li-Na Kang, Biao Xu

**Affiliations:** Department of Cardiology, Affiliated Drum Tower Hospital, Nanjing University School of Medicine, 321 Zhongshan Road, 210008 Nanjing, Jiangsu Province China; Department of Respiratory Medicine, Jinling Hospital, Nanjing University School of Medicine, Nanjing, China; Department of Radiology, Affiliated Drum Tower Hospital, Nanjing University School of Medicine, Nanjing, China

**Keywords:** PCSK9, Monoclonal antibody, Evolocumab, Alirocumab, Safety, Efficacy, LDL-C, Meta-analysis, Randomized controlled trials

## Abstract

**Background:**

Inhibition of proprotein convertase subtilisin/kexin type 9 (PCSK9) has been intensively studied to lower low-density lipoprotein cholesterol (LDL-C) levels. The purpose of this meta-analysis was to evaluate the safety and efficacy of anti-PCSK9 antibodies in randomized, controlled trials (RCTs).

**Methods:**

PubMed, EMBASE, CENTRAL databases, and recent conferences were searched. Safety outcomes were rates of common adverse events. Efficacy outcomes included percentages of LDL-C lowering and other lipid changes compared with placebo and ezetimibe, respectively.

**Results:**

Twenty-five RCTs encompassing 12,200 patients were included. The rates of common adverse events were firstly reported in our study by pooling together all evidence in RCTs, showing largely no significant difference between anti-PCSK9 antibodies and placebo (or ezetimibe), except that alirocumab was associated with reduced rates of death (relative risk (RR): 0.43, 95 % confidence interval (CI): 0.19 to 0.96, *P* = 0.04) and an increased rate of injection-site reactions (RR: 1.48, 95 % CI: 1.05 to 2.09, *P* = 0.02); evolocumab reduced the rate of abnormal liver function (RR: 0.43, 95 % CI: 0.20 to 0.93, *P* = 0.03), both compared with placebo. No significant difference in safety outcomes was detected between monthly 420 mg and biweekly 140 mg evolocumab treatments. Monthly 420 mg evolocumab treatment significantly reduced LDL-C by −54.6 % (95 % CI: −58.7 to −50.5 %) and by absolute −78.9 mg/dl (95 % CI: −88.9 to −68.9 mg/dl) versus placebo, and by −36.3 % (95 % CI: −38.8 to −33.9 %) versus ezetimibe, and increased high-density lipoprotein cholesterol (HDL-C) by 7.6 % (95 % CI: 5.7 to 9.5 %) versus placebo and 6.4 % (95 % CI: 4.3 to 8.4 %) versus ezetimibe. An equal or even greater change was observed following biweekly 140 mg administration. Significant and favorable changes were also detected in other lipids following evolocumab treatment. Biweekly 50 to 150 mg alirocumab lowered LDL-C by −52.6 % (95 % CI: −58.2 to −47.0 %) versus placebo, by −29.9 % (95 % CI: −32.9 to −26.9 %) versus ezetimibe, and increased HDL-C by 8.0 % (95 % CI: 4.2 to 11.7 %) versus placebo.

**Conclusions:**

Evolocumab and alirocumab were safe and well-tolerated from our most-powered analyses. Both antibodies substantially reduced the LDL-C level by over 50 %, increased the HDL-C level, and resulted in favorable changes in other lipids.

**Electronic supplementary material:**

The online version of this article (doi:10.1186/s12916-015-0358-8) contains supplementary material, which is available to authorized users.

## Background

Hypercholesterolemia is a major risk factor for cardiovascular disease (CVD) [1]. The introduction of statins has substantially reduced CVD events around the world and is recommended as first-line therapy for CVD management [2]. However, a necessity for other lipid-lowering (especially low-density lipoprotein cholesterol (LDL-C) lowering) agents still exists because some patients cannot tolerate statins due to adverse events, or cannot achieve intensive LDL-C lowering because of extremely high baseline LDL-C levels, or patients with very high risk of CVD events need more intensive lowering therapy [3].

The role of proprotein convertase subtilisin/kexin type 9 (PCSK9) in cholesterol regulation has been established since *PCSK9* mutations were first discovered in autosomal dominant hypercholesterolemia (ADH) in 2003 [4]. PCSK9 binds to LDL receptors (LDLR) and facilitates the degradation of LDLRs [5] and thus leads to LDL-C increase, indicating great therapeutic potential. Therefore, inhibiting PCSK9 by monoclonal antibodies [6, 7], small interfering RNA [8], and small molecule inhibitors [9] has been evaluated to lower LDL-C levels in human studies during the last few years. However, a comprehensive analysis of the safety of anti-PCSK9 antibodies is absent, and efficacy outcomes on lipid profiles are not uniformly consistent. Therefore, we performed a comprehensive review of the current available evidence to address the safety (to provide the exact rates of common adverse events) and the efficacy (to determine the exact extent of lipid changing effect) of anti-PCSK9 antibodies.

## Methods

### Literature search

We sought to identify all randomized, controlled trials (RCTs) evaluating the safety and efficacy of PCSK9 monoclonal antibodies. We searched PubMed, EMBASE, and the Cochrane Central Register of Controlled Trials (CENTRAL) from their inception to 6 October 2014, using the following search terms and key words: ‘AMG 145’, ‘evolocumab’, ‘SAR236553’, ‘REGN727x’, ‘SAR236553/REGN727’, ‘alirocumab’ and ‘PCSK9’. Reference lists of the identified reports and relevant reviews were manually checked. Major conference proceedings were searched to retrieve unpublished studies until the end of the American Heart Association (AHA) scientific sessions on 20 November 2014. We did not apply any restriction on languages.

### Study selection

Eligibility assessment was performed by two investigators (XZ and QZ). Studies were included if they: 1) were RCTs; 2) involved human subjects; 3) evaluated the safety and efficacy of an anti-PCSK9 antibody (evolocumab or alirocumab); and 4) reported mean differences with corresponding confidence intervals (CIs) or provided data necessary to calculate such. We did not restrict the type of study populations. We excluded animal studies, studies which were not randomized, and studies using other anti-PCSK9 antibodies, such as bococizumab, or PCSK9 inhibitors such as small interfering RNA because of the limited number of trials published regarding these PCSK9 inhibitors.

### Outcomes

The safety outcomes were rates of common adverse events, and the primary efficacy endpoints were percent and absolute reductions in LDL-C following anti-PCSK9 antibody treatment. Secondary outcomes included: 1) LDL-C reduction at 52 weeks follow-up for evolocumab; 2) other lipid profile changes stratified by treatment dosages and durations of follow-up.

### Data collection

Data were abstracted independently by two reviewers (XZ and QZ) using a standardized data extraction form. When there were disagreements, a third reviewer (LZ) checked the data. The following information was extracted: trial name/first author, year of publication, number of patients, duration of follow-up, age, gender, race, diabetes mellitus, coronary heart disease (CHD), PCSK9 level and all lipid profiles at baseline. Patient profile and background lipid-lowering therapy, treatments and doses in each study were also recorded. For safety endpoints, we extracted the number of events of interest and total number of patients in each group. For efficacy outcomes, as a priority, we extracted the mean differences and their corresponding 95 % CIs or standard errors (SEs) of anti-PCSK9 antibody versus placebo or ezetimibe for each lipid items. Alternatively, mean changes and 95 % CIs (or SEs) from baseline after either anti-PCSK9 antibody or placebo (or ezetimibe) treatments were extracted, thereafter mean differences of anti-PCSK9 antibody versus controls were calculated.

### Quality assessment

We followed the Cochrane Collaboration’s tool to assess the risk of bias of included trials. Random sequence generation (selection bias), allocation concealment (selection bias), blinding of participants and personnel (performance bias), blinding of outcome assessment (detection bias), incomplete outcome data (attrition bias), selective reporting (reporting bias) and other sources of bias were included in the assessment independently performed by two reviewers (QZ and LZ).

### Statistical analysis

For all efficacy outcomes, the mean differences following anti-PCSK9 treatment versus placebo or ezetimibe were pooled across studies using the DerSimonian-Laird random-effects models. Comparisons of anti-PCSK9 antibodies with placebo or ezetimibe were performed separately and stratified by dosages of antibodies. Adverse event rates were also pooled with random-effects models. Trials in which the endpoint was not detected in any of the treatment groups were excluded in the analysis of that endpoint. For studies in which only one of the groups had no event of interest, the estimate of treatment effect and its confidence interval were calculated after adding 0.5 to each cell of the 2 × 2 table for the trial [10, 11]. We used the *I*^*2*^ statistic to assess the consistency across studies, with 25 %, 50 %, and 75 % indicating low, moderate, and high degrees of heterogeneity respectively. Meanwhile, the χ2-based Q test was applied, and a *P* >0.10 suggests significant heterogeneity. Begg’s test and Egger’s test were performed to assess publication bias. Sensitivity analyses were carried out by omitting one study at one time to evaluate the consistency of the results.

In the LAPLACE-2 trial [7], efficacy data comparing evolocumab and ezetimibe were only reported in five subgroups stratified by background lipid-lowering therapies. We combined the results from these subgroups into a single group using the formulae recommended by the Cochrane Collaboration [12]. All analyses were conducted with the STATA version 11.0 software (STATA Corporation, College Station, TX, USA). The meta-analysis was in line with recommendations from the Preferred Reporting Items for Systematic Reviews and Meta-Analyses (PRISMA) statement (Additional file 1).

## Results

### Study selection and characteristics

**Fig. 1:**
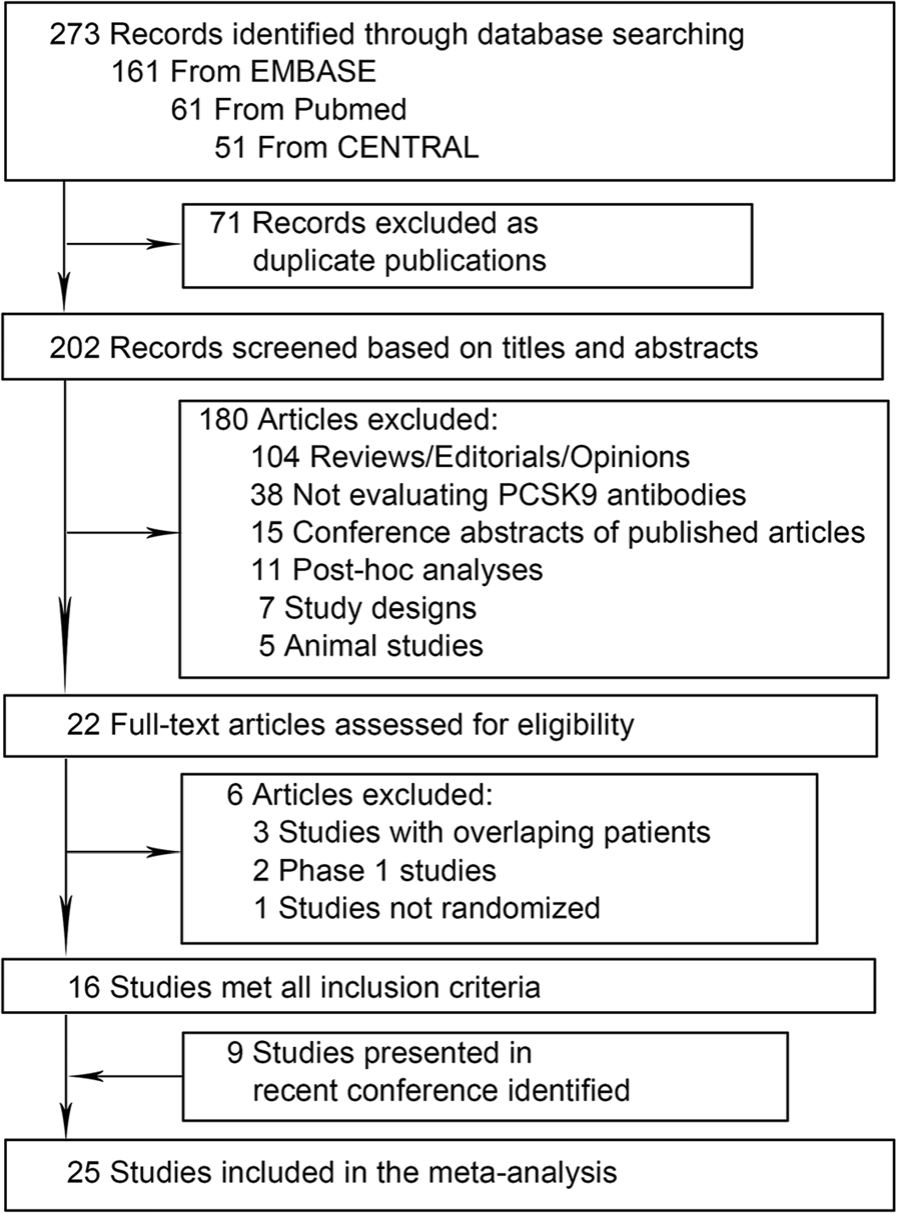
Flow diagram for study selection

Our systematic literature search yielded 273 studies. After excluding duplicate publications and studies which clearly did not meet the inclusion criteria based on titles and abstracts, 22 studies were retrieved for full-text review. Six studies were further excluded, in which one study was not RCT [13] and two were phase 1 trials with either non-constant dosage of anti-PCSK9 antibodies or with too few participants [14,15]. Nine additional studies were identified in the recent conference of the European Society of Cardiology (ESC) and AHA, and were included in the meta-analysis [16-22] (Fig. 1). During the revision process of this paper, two of the trials (ODYSSEY LONG TERM and ODYSSEY COMB II trials) included in our analysis as conference presentations [16, 18] from 2014 AHA and ESC scientific sessions were published [23, 24]. Thus, 25 studies were included, encompassing a total of 12,200 patients. Twelve trials were conducted using anti-PCSK9 antibody evolocumab (AMG 145) [7, 25–35], and 13 were on alirocumab (SAR236553/REGN727) [16–22, 36–39]. The OSLER study was carried out based on participants from four parent trials (GAUSS, MENDEL, LAPLACE-TIMI 57, and RUTHERFORD) and was followed up for 52 weeks [30].

Baseline characteristics of individual trials are shown in Table 1 and Table S1 and S2 (in Additional file 2). Several studies did not report age, lipids or PCSK9 level in the overall population. Therefore, we presented these characteristics in control populations (placebo or ezetimibe) in these studies given the significantly similar baseline values between the anti-PCSK9 treatment group and controls. All randomized trials included were published or presented in major conferences between 2012 and 2014. The mean age ranged from 31 to 62 years, and the percentage of women from 37 to 74; over 80 % of the patients were white. Regarding evolocumab, all trials were followed up for 12 weeks, except the OSLER and DESCARTES trials [25, 30], which were followed up for 52 weeks. With regard to alirocumab, most trials were followed up for 24 weeks except three phase 2 trials which were followed-up for 8 to 12 weeks [36, 37, 39]. All included RCTs had a low risk of bias, as detailed in Table S3 (in Additional file 2).

**Table 1:** Baseline characteristics of included randomized trials

Trial/first author	Year	Number	Follow-up, weeks	Age, years	Women, number (%)	LDL-C, mmol/L	Total-C, mmol/L	HDL-C, mmol/L	Free PCSK9, nmol/L	Statin use, number (%)	Ezetimibe use, number (%)	CHD, number (%)	DM, number (%)	Patient profile and background lipid-lowering therapy
RUTHERFORD	2012	167	12	50 (13)	79 (47)	4.0 (1.1)	6.1 (1.3)	1.3 (0.4)	8.3 (2.4)	150 (89)	108 (64)	35 (21)	NA	HeFH with LDL-C ≥2.6 mmol/L. Statin ± ezetimibe
LAPLACE-TIMI 57	2012	629	12	62 (55, 67)	320 (51)	3.2 (0.7)	5.2 (0.9)	1.4 (0.4)	6.2 (1.7)	627 (99)	57 (9)	187 (30)	103 (16)	LDL-C ≥2.2 mmol/L and triglycerides ≤4.5 mmol/L. Statin ± ezetimibe
GAUSS	2012	157	12	62 (8)	100 (64)	5.0 (1.3)	7.3 (1.4)	1.5 (0.5)	5.3 (1.4)	0	64 (40)	21 (13.4)	NA	LDL-C ≥2.6 mmol/L with diagnosed CHD or risk equivalent; ≥3.4 mmol/L without CHD or risk equivalent and 2 or more risk factors, or ≥4.1 mmol/L without CHD or risk equivalent and with 1 or 0 risk factors. No/low-dose statin or statin-intolerance
MENDEL	2012	406	12	51 (12)	267 (66)	3.7 (0.6)	5.7 (0.8)	1.4 (0.4)	4.8 (1.2)	0	45 (11)	0	1 (0.2)	LDL-C ≥2.6 and <4.9 mmol/L and triglycerides ≤4.5 mmol/L, and a 10 year Framingham risk score for coronary heart disease of up to 10 %. No background anti-lipid therapy
YUKAWA	2014	307	12	62 (10)	114 (37)	3.7 (0.5)	5.8 (0.6)	1.4 (0.3)	5.6 (1.8)	307 (100)	NA	77 (25)	117 (38)	LDL-C ≥3.0 mmol/L and triglycerides ≤4.5 mmol/L high risk for cardiovascular events. Statin ± ezetimibe
MENDEL-2	2014	614	12	54 (10)	423 (69)	3.6 (0.5)	NA	1.5 (1.1, 2.0)	3.9 (1.2)	0	154 (25)	0	1 (0.1)	LDL-C ≥2.6 and <4.9 mmol/L, triglycerides ≤4.5 mmol/L, and 10-year Framingham coronary heart disease risk scores ≤ 10 %. No lipid regulating drugs within 3 months
LAPLACE-2	2014	1896	12	60 (10)	868 (46)	2.8 (1.0)	4.9 (1.1)	1.4 (0.4)	4.9 (1.6)	1327 (70)	NA	427 (23)	293 (16)	LDL-C ≥3.9 mmol/L (no statin at screening), ≥2.6 mmol/L (nonintensive statin at screening), or ≥2.1 mmol/L (intensive statin at screening) and triglyceride ≤4.5 mmol/L
GAUSS-2	2014	307	12	62 (10)	141 (46)	5.0 (1.5)	NA	1.3 (0.5)	4.4 (1.7)	55 (18)	NA	NA	62 (20)	LDL-C ≥ 2.6 mmol/L and triglycerides ≤4.5 mmol/L. No/low-dose statin or statin-intolerance
DESCARTES	2014	901	52	57 (10)	471 (52)	2.7 (0.6)	4.6 (0.7)	1.4 (0.4)	6.7 (2.2)	790 (88)	189 (21)	136 (15)	104 (12)	LDL-C ≥1.9 mmol/L and triglycerides ≤4.5 mmol/L. Statin ± ezetimibe
OSLER	2014	1104	52	57 (12)	610 (55)	3.7 (1.0)	5.8 (1.2)	1.4 (0.4)	5.8 (2.1)	691 (63)	NA	210 (19)	109 (10)	From parent studies (RUTHERFORD, LAPLACE-TIMI 57, GAUSS, MENDEL)
TESLA	2014	49	12	31 (13)	24 (49)	9.0 (3.5)	NA	1.0 (0.3)	9.0 (2.7)	49 (100)	45 (92)	21 (43)	NA	Homozygous familial hypercholesterolaemia, LDL-C ≥3.4 mmol/L. Statin ± ezetimibe
RUTHERFORD-2	2014	329	12	51 (14)	139 (42)	3.9 (1.0)	NA	1.4 (0.4)	6.0 (1.7)	329 (100)	204 (62)	103 (31)	NA	HeFH patients ≥2.6 mmol/L. Statin ± ezetimibe
McKenney	2012	183	12	57 (10)	96 (53)	3.4 (0.7)	5.4 (0.7)	1.3 (0.3)	NA	NA	NA	10 (6)	22 (12)	LDL-C ≥2.6 mmol/L on stable-dose atorvastatin for ≥6 weeks
Stein	2012	77	12	53 (10)	30 (39)	3.9 (0.9)	6.1 (1.0)	1.4 (0.3)	NA	77 (100)	55 (71)	32 (42)	3 (4)	HeFH and LDL-C ≥2.6 mmol/L. Statin ± ezetimibe
Roth	2012	92	8	57 (10)	55 (60)	3.2 (0.5)	5.2 (0.7)	1.4 (0.4)	NA	92 (100)	NA	3 (3)	14 (15)	LDL-C ≥2.6 mmol/L on stable-dose atorvastatin for ≥7 weeks
ODYSSEY COMBO II	2014	720	24	61 (9)	530 (74)	2.7 (0.9)	NA	NA	NA	719 (99.9)	NA	580 (81)	221 (31)	LDL-C ≥1.8 mmol/L (history of CVD) or ≥2.6 mmol/L (no history of CVD) High CV-risk patients on max-tolerated statin
ODYSSEY FH I	2014	486	24	52 (12)	212 (55)	3.7 (1.2)	NA	NA	NA	486 (100)	277 (57)	225 (46)	56 (12)	HeFH, inadequately controlled on maximally tolerated stable statin therapy with or without other LLT
ODYSSEY FH II	2014	249	24	53 (13)	118 (47)	3.5 (1.1)	NA	NA	NA	249 (100)	165 (66)	88 (35)	10 (4)	HeFH, inadequately controlled on maximally tolerated stable statin therapy with or without other LLT
ODYSSEY LONG TERM	2014	2341	24	61 (10)	884 (38)	3.2 (1.1)	NA	NA	NA	2339 (99.9)	334 (14)	1607 (69)	809 (35)	HeFH or High-CV risk patients LDL-C ≥1.8 mmol/L on max-tolerated statin therapy with or without other LLT
ODYSSEY MONO	2014	103	24	60 (5)	48 (47)	3.6 (0.6)	5.8 (0.8)	1.6 (0.5)	NA	0	0	NA	4 (4)	LDL-C ≥2.6 and <4.9 mmol/L, 10-year risk of fatal cardiovascular events ≥1 % and ≤5 %
ODYSSEY ALTERNATIVE	2014	251	24	63 (10)	114 (45)	5.0 (1.8)	NA	1.3 (0.4)	NA	NA	NA	118 (47)	60 (24)	Statin intolerant patients (by medical history) with LDL-C ≥70 mg/dl (very-high CV risk) or ≥ 100 mg/dl (moderate/high risk)
ODYSSEY COMBO I	2014	316	24	63 (9)	108 (34)	2.7 (0.9)	NA	1.3 (0.3)	NA	315 (100)	26 (8)	247 (78)	136 (43)	High CV risk on maximally tolerated statin with or without other LLT (LDL-C ≥70 mg/dl manifest CVD; or LDL-C ≥100 mg/dl with DM and other risk factors or CKD)
ODYSSEY HIGH FH	2014	107	24	52 (11)	50 (47)	5.2 (1.1)	NA	NA	NA	107 (100)	26 (24)	53 (50)	15 (14)	HeFH inadequately controlled on maximally tolerated stable statin therapy with or without other LLT (LDL-C ≥160 mg/dl)
ODYSSEY OPTION I	2014	205	24	66 (9)	75 (36)	2.6 (0.8)	NA	NA	NA	182 (100)	NA	NA	NA	Patients with prior CVD + LDL-C ≥70 mg/dl, or CV risk factors + LDL-C ≥100 mg/dl
ODYSSEY OPTION II	2014	204	24	60 (10)	88 (43)	2.7 (1.1)	NA	NA	NA	175 (100)	NA	NA	NA	Patients with prior CVD + LDL-C ≥70 mg/dl, or CV risk factors + LDL-C ≥100 mg/dl

Data are mean (SD), mean (SE), number (%), or median (IQR); lipid profiles are mean (SE) if not indicated; age is mean (SD). DESCARTES, the Durable Effect of PCSK9 Antibody Compared with Placebo Study trial; GAUSS, the Goal Achievement after Utilizing an anti-PCSK9 antibody in Statin Intolerant Subjects trial; LAPLACE-TIMI 57, the LDL-C Assessment With PCSK9 Monoclonal Antibody Inhibition Combined With Statin Therapy (LAPLACE)–Thrombolysis in Myocardial Infarction (TIMI) 57 trial; MENDEL, Monoclonal Antibody Against PCSK9 to Reduce Elevated LDL-C in Subjects Currently Not Receiving Drug Therapy for Easing Lipid Levels trial; OSLER, the Open Label Study of Long Term Evaluation Against LDL-C trial; RUTHERFORD, The Reduction of LDL-C With PCSK9 Inhibition in Heterozygous Familial Hypercholesterolemia Disorder trial; TESLA, The Trial Evaluating PCSK9 Antibody in Subjects with LDL Receptor Abnormalities; YUKAWA, the StudY of LDL-Cholesterol Reduction Using a Monoclonal PCSK9 Antibody in Japanese Patients With Advanced Cardiovascular Risk trial

CHD, coronary heart disease; CVD, cardiovascular disease; DM, diabetes mellitus; HDL-C, high-density lipoprotein (HDL) cholesterol; HeFH, heterozygous familial hypercholesterolemia IQR, interquartile range; LDL-C, low-density lipoprotein (LDL) cholesterol; LLT, lipid-lowering therapy; NA, not applicable; PCSK9, proprotein convertase subtilisin/kexin type 9; SD, standard deviation; SE, standard error; Total-C, total cholesterol

### Safety outcomes of evolocumab

The pooled estimate for overall incidence of any treatment emergent adverse events (TEAEs) was 52.2 % (95 % CI: 44.8 to 59.7 %) at 12 weeks follow-up, which was not significantly different from placebo (pooled rate: 45.2 %; 95 % CI: 40.6 to 49.8 %) (relative risk (RR): 1.07, 95 % CI: 0.95 to 1.21) or ezetimibe (pooled rate: 54.7 %; 95 % CI: 41.3 to 68.0 %) (RR: 0.92, 95 % CI: 0.84 to 1.01, Table 2). Serious TEAE occurred in 1.9 % patients, TEAEs leading to discontinuation in 1.6% patients at 12 weeks following evolocumab treatment. Only 1 in 3,068 patients died at 12 weeks follow-up and 3 in 1,335 patients at 52 weeks follow-up, which were all similar to control groups (Table 2). Sixteen in 2,797, 12 in 2,797, and 20 in 2,287 patients developed creatine kinase (CK) elevations greater than five times the upper limit of normal (ULN), elevations in aspartate aminotransferase/alanine aminotransferase (AST/ALT) levels greater than three times the ULN, and adjudicated cardiovascular events respectively. Patients receiving evolocumab had a lower risk of developing abnormal liver function (AST/ALT greater than three times ULN) than those receiving placebo at 12-week follow-up (RR: 0.43, 95 % CI: 0.20 to 0.93, *P* = 0.03), but the difference did not maintain at 52-week follow-up. The pooled incidence of musculoskeletal and connective-tissue disorders was 9.8 % (95 % CI: 4.1 to 15.4 %), which was not significantly different with placebo (pooled rate: 7.1 %; 95 % CI: 1.6 to 12.6 %) (RR 1.08, 95 % CI: 0.70 to 1.67) or ezetimibe (pooled rate: 6.1 %; 95% CI: 0.7 to 11.5 %) (RR 1.10, 95 % CI: 0.61 to 2.00). Injection-site reactions occurred in 2.2 % of patients. No significant difference in any reported adverse event was found between monthly 420 mg and biweekly 140 mg administration at 12 weeks follow-up (Table 3). The event rates at 52-week follow-up following evolocumab are also reported in Table 2.

**Table 2:** Adverse event rates at 12- and 52-week follow-up following evolocumab, placebo or ezetimibe treatments

Safety endpoints	Evolocumab (12 Week)		Placebo (12 Week)		Evolocumab versus Placebo (12 Week)		Ezetimibe (12 Week)		Evolocumab versus Ezetimibe (12 Week)		Evolocumab (52 Week)
	Pooled event rate (95 % CI)	Event/Total	Pooled event rate (95 % CI)	Event/Total	RR (95 % CI)	*P* value	Pooled event rate (95 % CI)	Event/Total	RR (95 % CI)	*P* value	Event/Total
TEAE (Any)	52.2 (44.8, 59.7)	1472/3068	45.2 (40.6, 49.8)	534/1240	1.07 (0.95, 1.21)	0.260	54.7 (41.3, 68.0)	278/554	0.92 (0.84, 1.01)	0.074	78.4 (1047/1335)
TEAE (Serious)	1.9 (1.4, 2.4)	64/3068	1.2 (0.5, 1.9)	23/1240	0.96 (0.60, 1.55)	0.876	0.9 ( 0.3, 1.6)	7/554	1.35 (0.61, 3.00)	0.458	6.4 (85/1335)
Leading to discontinuation	1.6 (0.9, 2.4)	56/3068	1.1 (0.4, 1.8)	21/1240	0.78 (0.46, 1.32)	0.354	3.5 (1.0, 6.0)	24/554	0.68 (0.42, 1.11)	0.127	3.0 (40/1335)
Death	NA	1/3068	NA	1/1240	NA	NA	NA	0/554	NA	NA	0.2 (3/1335)
CK >5 ULN	0.5 (0.1, 0.8)	16/2797	0.5 (0.2, 0.8)	8/1150	0.57 (0.21, 1.51)	0.258	0.5 (0, 0.8)	4/509	0.55 (0.17, 1.81)	0.325	1.0 (14/1335)
ALT or AST > 3 ULN	0.2 (0.1, 0.4)	12/2797	0.8 (0.3, 1.2)	13/1150	0.43 (0.20, 0.93)	0.033	0.7 (0.1, 1.3)	4/509	0.43 (0.14, 1.34)	0.147	1.3 (18/1335)
Adjudicated cardiovascular events	0.6 (0.2, 1.1)	20/2287	0.5 (0.1, 0.9)	7/1014	1.07 (0.41, 2.76)	0.892	0.9 (0, 1.9)	2/266	0.50 (0.12, 2.13)	0.346	1.1 (15/1335)
Musculoskeletal and connective-tissue disorders	9.8 (4.1, 15.4)	144/1397	7.1 (1.6, 12.6)	39/508	1.08 (0.70, 1.67)	0.738	6.1 (0.7, 11.5)	13/231	1.10 (0.61, 2.00)	0.751	9.2 (68/736)
Back pain	2.6 (1.7, 3.4)	56/2208	1.8 (0.7, 2.8)	21/912	1.05 (0.53, 2.11)	0.883	2.5 (0.9, 4.1)	8/298	0.72 (0.34, 1.54)	0.4	6.4 (85/1335)
Arthralgia	1.7 (1.0, 2.5)	35/1862	1.7 (0.9, 2.6)	14/803	1.04 (0.56, 1.93)	0.912	1.5 (0.2, 2.9)	4/266	0.97 (0.35, 2.64)	0.945	5.7 (76/1335)
Muscle spasms	1.9 (0.7, 3.2)	45/2193	1.3 (0.5, 2.0)	11/803	1.02 (0.42, 2.49)	0.963	2.5 (0.7, 4.3)	13/400	0.67 (0.30, 1.50)	0.335	2.3 (14/599)
Myalgia	3.5 (1.5, 5.6)	48/1382	1.0 (0.2, 1.8)	5/399	1.13 (0.37, 3.43)	0.833	5.0 (0.6, 9.4)	23/333	0.68 (0.30, 1.56)	0.364	4.0 (24/599)
Headache	3.4 (2.2, 4.6)	86/2830	2.6 (1.5, 3.7)	34/1122	0.81 (0.53, 1.24)	0.331	2.8 (1.2, 4.4)	20/554	0.94 (0.57, 1.55)	0.798	4.0 (24/599)
Injection-site reactions	2.2 (1.3, 3.1)	64/2831	1.7 (0.9, 2.5)	26/1184	1.06 (0.67, 1.67)	0.816	2.0 (0.4, 3.6)	13/522	1.02 (0.54, 1.93)	0.955	5.4 (72/1335)
Gastrointestinal disorders	5.6 (2.7, 8.4)	118/1620	5.3 (1.9, 8.7)	33/580	1.09 (0.68, 1.75)	0.73	6.8 (0.1, 13.4)	18/301	0.81 (0.47, 1.39)	0.441	6.3 (38/599)
Nasopharyngitis	6.2 (3.6, 8.8)	115/1746	4.2 (2.1, 6.3)	28/580	1.39 (0.93, 2.08)	0.11	4.8 (1.6, 8.0)	18/333	0.54 (0.30, 1.15)	0.113	11.5 (153/1335)
Influenza	1.7 (0.5, 2.8)	27/1220	2.0 (0, 4.3)	9/317	0.89 (0.38, 2.07)	0.792	2.1 (0.1, 4.0)	5/179	0.34 (0.10, 1.18)	0.090	7.3 (97/1335)
Upper respiratory tract infection	4.2 (2.5, 5.9)	43/1015	2.9 (0.3, 5.6)	12/317	1.01 (0.54, 1.90)	0.964	5.3 (0, 14.4)	5/77	0.74 (0.22, 2.50)	0.624	8.5 (113/1335)

ALT, alanine aminotransferase; AST, aspartate aminotransferase; CI, confidence interval; CK, creatine kinase; NA, not applicable; TEAE, treatment emergent adverse event; ULN, upper limit of normal

**Table 3:** Adverse event rates at 12-week follow-up following different dosages of evolocumab treatments

Safety endpoints	420 mg Q4W (12 Week)		140 mg Q2W (12 Week)		420 mg Q4W versus 140 mg Q2W (12 Week)	
	Pooled event rate (95 % CI)	Event/Total	Pooled event rate (95 % CI)	Event/Total	RR (95 % CI)	*P* value
TEAE (Any)	52.1 (42.9, 61.3)	565/1228	52.0 (43.1, 60.9)	496/1095	1.01 (0.92, 1.10)	0.873
TEAE (Serious)	1.4 (0.8, 1.9)	23/1228	2.5 (1.6, 3.3)	30/1095	0.69 (0.39, 1.23)	0.214
Leading to discontinuation	1.4 (0.6, 2.2)	26/1228	1.8 (0.8, 2.9)	26/1095	0.94 (0.54, 1.64)	0.835
Death	NA	0/1228	NA	1/1095	NA	NA
CK >5 ULN	0.5 (0.1, 0.9)	8/1183	0.1 (0, 0.3)	2/1050	1.58 (0.44, 5.75)	0.484
ALT or AST >3 ULN	0.4 (0.2, 0.7)	5/1183	0.5 (0.1, 0.8)	5/1050	0.70 (0.20, 2.44)	0.573
Adjudicated cardiovascular events	1.1 (0.2, 2.0)	3/288	1.5 (0.3, 2.6)	6/285	0.56 (0.17, 1.92)	0.36
Musculoskeletal and connective-tissue disorders	3.9 (1.0, 6.9)	21/421	8.0 (3.4, 12.5)	30/386	0.63 (0.29, 1.34)	0.227
Back pain	3.6 (1.0, 6.3)	20/830	2.4 (1.4, 3.5)	20/788	1.09 (0.42, 2.87)	0.86
Arthralgia	1.8 (0.9, 2.7)	13/687	1.5 (0.6, 2.3)	10/678	1.27 (0.56, 2.87)	0.568
Muscle spasms	2.0 (0.5, 3.5)	18/823	1.4 (0.4, 2.5)	15/780	1.10 (0.56, 2.20)	0.776
Myalgia	1.3 (0, 2.7)	11/414	2.3 (0.4, 4.2)	12/378	0.91 (0.40, 2.06)	0.82
Headache	3.1 (1.7, 4.4)	40/1142	2.7 (1.3, 4.2)	26/1043	1.40 (0.84, 2.33)	0.202
Injection-site reactions	2.0 (0.9, 3.1)	18/577	2.4 (0.8, 4.0)	19/540	0.94 (0.50, 1.78)	0.853
Gastrointestinal disorders	4.9 (2.3, 7.5)	35/580	5.4 (2.5, 8.2)	28/488	1.13 (0.68, 1.87)	0.637
Nasopharyngitis	4.9 (2.5, 7.4)	36/613	4.1 (1.7, 6.6)	24/488	1.10 (0.63, 1.89)	0.744
Influenza	1.2 (0.1, 2.4)	9/350	2.5 (0.4, 5.4)	8/225	0.19 (0.03, 1.10)	0.064
Upper respiratory tract infection	4.6 (2.2, 6.9)	14/247	4.8 (1.2, 8.4)	6/123	1.30 (0.46, 3.70)	0.621

ALT, alanine aminotransferase; AST, aspartate aminotransferase; CI, confidence interval; CK, creatine kinase; NA, not applicable; RR, relative risk; TEAE, treatment emergent adverse event; ULN, upper limit of normal

### Safety outcomes of alirocumab

Three phase 2 studies reported safety outcomes at 8 to 12 weeks, while other phase 3 studies reported either at 24-week or 52-week follow-up. Safety profiles were pooled together in all trials. Any TEATs happened in 71.7 % (95 % CI: 67.7 to 75.6 %) patients following alirocumab treatment, mirrored to those with placebo (68.4 %, 95 % CI: 58.7 to 76.2 %) (RR: 1.00, 95 % CI: 0.92 to 1.10) or ezetimibe treatment (70.1 %, 95 % CI: 62.9 to 77.4 %) (RR: 1.01, 95 % CI: 0.96 to 1.07, Table 4). TEAEs which were serious or led to discontinuation occurred in 8.6 % and 4.8 % of patients, respectively. Fifteen in 3,363, 11 in 992, and 7 in 862 died following alirocumab, placebo or ezetimibe treatments, respectively, showing a lower rate in alirocumab compared with placebo (RR: 0.43, 95 % CI: 0.19 to 0.96, *P* = 0.04), but not ezetimibe (RR: 0.48, 95 % CI: 0.16 to 1.45, *P* = 0.19). CK greater than three times ULN, ALT/AST greater than three times ULN, and adjudicated cardiovascular events were detected in 2.0 %, 0.9 %, and 2.6 % of patients, respectively (Table 4). A trend toward a lower rate of serum CK level elevation was observed in alirocumab group than placebo group (RR: 0.72, 95 % CI: 0.52 to 1.01, *P* = 0.06). Musculoskeletal and connective-tissue disorders occurred in 16.7 % patients. A higher rate of injection-site reactions was detected following alirocumab administration (pooled rate: 6.0 %, 95 % CI: 3.8 to 8.2 %) than placebo (pooled rate: 3.7 %, 95 % CI: 2.5 to 4.8 %) (RR: 1.48, 95 % CI: 1.05 to 2.09, *P* = 0.02). Neurocognitive disorders were observed in 0.6 % alirocumab-treated patients. Still, all other reported adverse events rates did not differ significantly between alirocumab and placebo/ezetimibe treatments.

**Table 4:** Adverse event rates following alirocumab, placebo or ezetimibe treatments

Safety endpoint	Alirocumab		Placebo		Alirocumab versus Placebo		Ezetimibe		Alirocumab versus Ezetimibe	
	Pooled event rate (95 % CI)	Event/Total	Pooled event rate (95 % CI)	Event/Total	RR (95 % CI)	*P* value	Pooled event rate (95 % CI)	Event/Total	RR (95 % CI)	*P* value
TEAE (Any)	71.7 (67.7, 75.6)	2561/3425	68.4 (58.7, 78.2)	783/1007	1.00 (0.92, 1.10)	0.938	70.1 (62.9, 77.4)	605/862	1.01 (0.96, 1.07)	0.615
TEAE (Serious)	8.6 (4.5, 12.8)	455/3363	9.3 (1.2, 17.4)	158/992	0.94 (0.79, 1.12)	0.47	8.5 (4.1, 12.8)	91/862	1.03 (0.81, 1.31)	0.815
Leading to discontinuation	4.8 (2.7, 6.9)	187/3363	4.6 (2.1, 7.1)	56/992	1.07 (0.78, 1.47)	0.667	7.9 (3.9, 12.0)	69/862	0.83 (0.38, 1.83)	0.645
Death	0.5 (0.3, 0.7)	15/3363	1.2 (0.5, 1.8)	11/992	0.43 (0.19, 0.96)	0.04	0.5 (0.1, 1.0)	7/862	0.48 (0.16, 1.45)	0.192
CK >3 ULN	2.0 (1.0, 3.1)	114/3415	3.9 (2.0, 5.8)	54/1003	0.72 (0.52, 1.01)	0.059	2.4 (1.1, 3.7)	28/855	0.75 (0.46, 1.24)	0.261
ALT or AST >3 ULN	0.9 (0.5, 1.3)	25/1869	1.3 (0.2, 2.4)	2/218	0.95 (0.26, 3.47)	0.94	0.5 (0.2, 0.9)	4/858	1.91 (0.75, 4.88)	0.176
Musculoskeletal and connective-tissue disorders	16.7 (5.9, 27.6)	536/2450	17.3 (3.8, 30.7)	235/865	1.00 (0.87, 1.14)	0.967	22.3 (0, 46.5)	74/416	0.80 (0.60, 1.05)	0.108
Injection-site reactions	6.0 (3.8, 8.2)	225/3425	3.7 (2.5, 4.8)	42/1007	1.48 (1.05, 2.09)	0.024	3.0 (1.1, 4.9)	35/862	1.30 (0.88, 1.92)	0.194
Adjudicated cardiovascular events	2.6 (1.3, 3.9)	109/3130	3.2 (1.3, 5.0)	38/930	0.94 (0.64, 1.39)	0.768	1.2 (0.5, 1.9)	15/811	1.29 (0.71, 2.36)	0.405
Nervous system disorders	9.3 (4.2, 14.5)	338/2813	6.6 (0.0, 15.9)	145/865	0.97 (0.81, 1.17)	0.776	6.2 (4.2, 8.2)	34/536	0.85 (0.56, 1.30)	0.461
Gastrointestinal disorders	16.4 (9.4, 23.4)	332/1845	13.3 (5.1, 21.5)	158/865	1.01 (0.57, 1.80)	0.964	9.8 (1.6, 8.0)	5/51	1.77 (0.63, 4.91)	0.276
Neurocognitive disorders	0.6 (0.2, 1.1)	27/2923	0.6 (0.1, 1.0)	6/930	1.03 (0.23, 4.60)	0.97	1.3 (0.5, 2.1)	8/609	0.65 (0.22, 1.91)	0.431

ALT, alanine aminotransferase; AST, aspartate aminotransferase; CI, confidence interval; CK, creatine kinase; RR, relative risk; TEAE, treatment emergent adverse event; ULN, upper limit of normal

### Primary efficacy outcomes of evolocumab

**Fig. 2:**
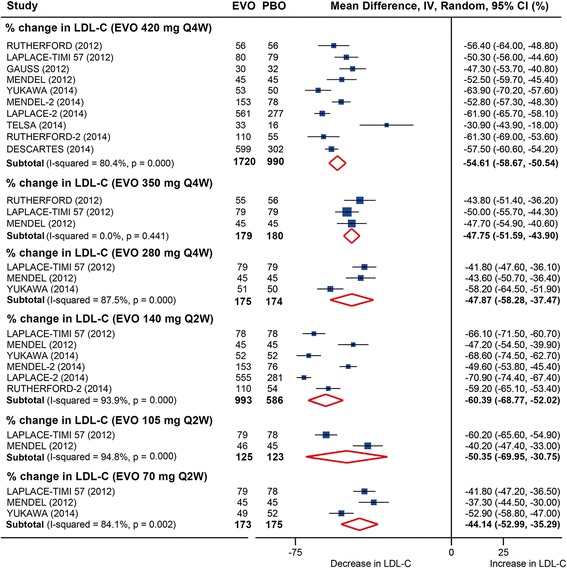
Pooled analysis for percent changes in LDL-C following evolocumab treatments stratified by dosages versus placebo at 12 weeks follow-up. EVO, evolocumab; PBO, placebo. LDL-C, low-density lipoprotein cholesterol

All six dosages of evolocumab significantly decreased LDL-C level at 12 weeks follow-up, with the greatest reductions achieved in monthly 420 mg evolocumab (mean reduction: −54.6 %, 95 % CI: −58.7 to −50.5 %) and biweekly 140 mg evolocumab (mean reduction: −60.4 %, 95 % CI: −68.8 to −52.0 %) versus placebo (Fig. 2 and Additional file 2: Table S4). There was significant heterogeneity in both comparisons (*I*^*2*^ = 80.4 % and 93.9 %, respectively). Biweekly administration of 140 mg evolocumab led to even greater reduction than 420 mg monthly treatment, both of which reduced the LDL-C level by over 50 %. The effect is likely dose-dependent with the same frequency of administration. Likewise, in absolute level changes, 420 mg monthly and 140 mg biweekly dosing lowered LDL-C by −78.9 mg/dl (95 % CI: −88.9 to −68.9 mg/dl) and −81.6 mg/dl (95 % CI: −92.0 to −71.1 mg/dl), respectively (Additional file 2: Figure S1 and Table S4).

**Fig. 3:**
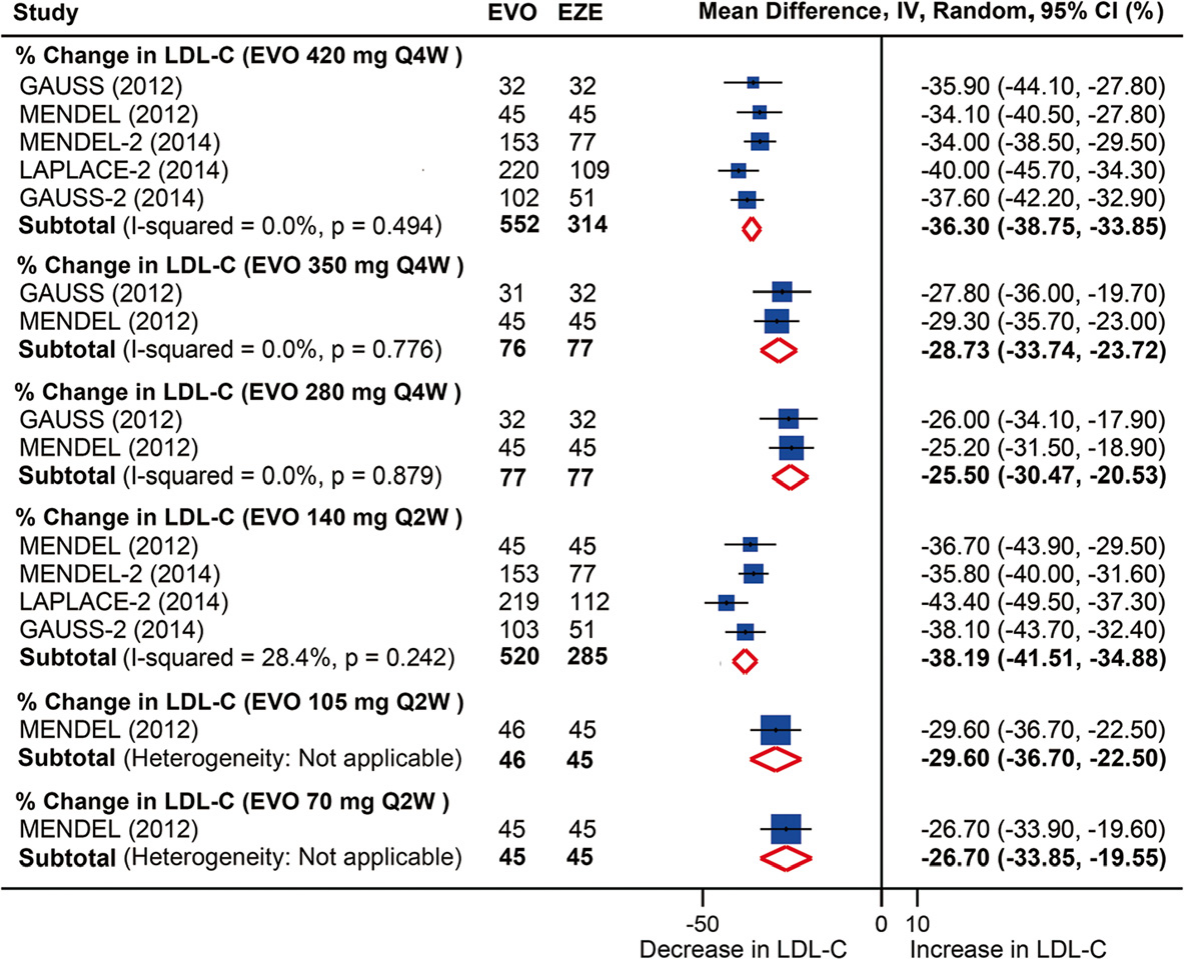
Pooled analysis for percent changes in LDL-C following evolocumab treatments stratified by dosages versus ezetimibe at 12 weeks follow-up. EVO, evolocumab; EZE, ezetimibe. LDL-C, low-density lipoprotein cholesterol

Compared with ezetimibe, significant lowering of LDL-C also occurred in all evolocumab dosages at week 12. Monthly 420 mg and biweekly 140 mg evolocumab administration reduced LDL-C level by −36.3 % (95 % CI: −38.8 to −33.9 %) and −38.2 % (95 % CI: −41.5 to −34.5 %), respectively, versus ezetimibe (Fig. 3 and Additional file 2: Table S4). No significant heterogeneity was detected in the comparisons (*I*^*2*^ = 0 and 28.4 %, respectively). Fewer studies reported absolute changes of LDL-C level versus ezetimibe; meta-analyses of these studies demonstrated largely similar but less remarkable results compared with those versus placebo.

LDL-C percent and absolute changes at the mean of weeks 10 and 12 following evolocumab treatments versus placebo or ezetimibe were all significant and similar to those changes at week 12 (Additional file 2: Table S5).

### Other efficacy outcomes of evolocumab

**Fig. 4:**
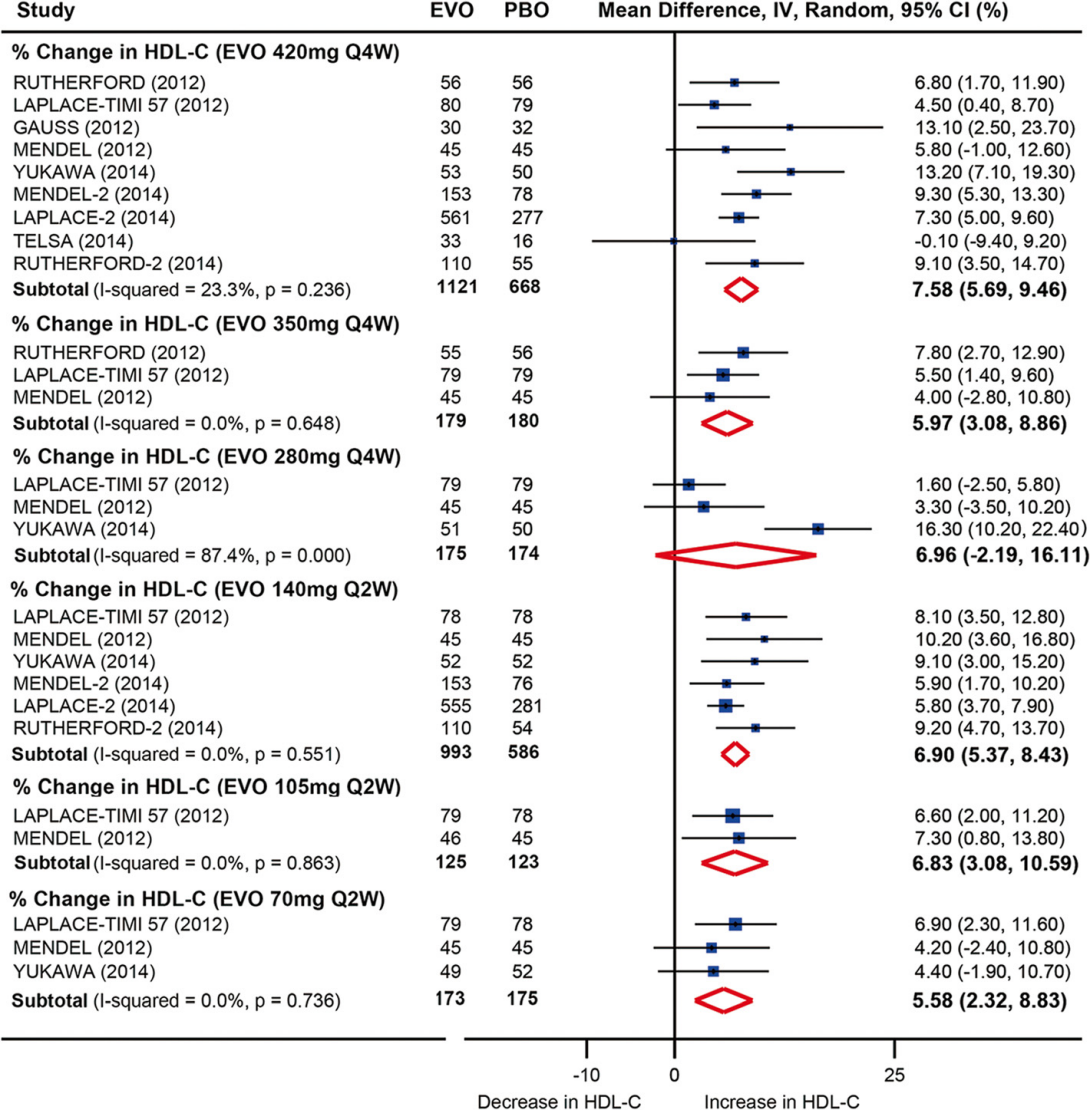
Pooled analysis for percent changes in HDL-C following evolocumab treatments stratified by dosages versus placebo at 12 weeks follow-up. EVO, evolocumab; PBO, placebo. HDL-C, high-density lipoprotein cholesterol

All dosages except monthly 280 mg evolocumab treatment significantly increased high-density lipoprotein cholesterol (HDL-C) levels at week 12 compared with placebo. The HDL-C level was increased by 7.6 % (95 % CI: 5.7 to 9.5 %) and 6.9 % (95 % CI: 5.4 to 8.4 %) by monthly 420 mg and biweekly 140 mg evolocumab treatment, respectively (Fig. 4 and Additional file 2: Table S6). No significant heterogeneity was detected in the comparisons (*I*^*2*^ = 23.3 % and 0, respectively). These two dosages of evolocumab also increased the HDL-C level compared with ezetimibe by 6.4 % (95 % CI: 4.3 to 8.4 %) and 7.2 % (95 % CI: 4.4 to 10.0 %), with no significant heterogeneity (*I*^*2*^ = 0 and 32.2 %, respectively).

Compared with placebo, all dosages of evolocumab generated significant reductions of total cholesterol (TC), TC/HDL-C, non-HDL-C and very low-density lipoprotein cholesterol (VLDL-C), which were decreased by monthly 420 mg evolocumab by −36.7 % (95 % CI: −38.9 to −34.4 %), −41.3 % (95 % CI: −45.7 to −36.9 %), −52.1 % (95 % CI: −55.1 to −49.1 %), and −22.8 % (95 % CI: −27.5 to −18.0 %), respectively at week 12, with low to modest levels of heterogeneity (*I*^*2*^ = 38.0 %, 64.7 %, 57.9 % and 6.6 %, respectively) (Table 5 and Additional file 2: Figures S2 to S5, Tables S7 to S10). Similar results were detected following biweekly 140 mg evolocumab treatment.

**Table 5:** Additional lipid efficiency outcomes following evolocumab treatments stratified by dosages versus placebo at 12-week follow-up

Endpoints	Evolocumab dosages	Mean Difference, % (95 % CI)	Test for overall effect		Number of studies	Number of individuals		Heterogeneity		Publication bias	
			*Z*	*P* value		Evolocumab	Placebo	*I* ^*2*^	*P* value	*P*_Begg	*P*_Egger
TC	420 mg Q4W	−36.66 (−38.93, −34.39)	31.60	0.000	6	539	825	38.0 %	0.153	1.000	0.980
	140 mg Q2W	−40.48 (−45.33, −35.62)	16.35	0.000	4	456	730	85.3 %	0.000	0.734	0.552
HDL-C	420 mg Q4W	7.58 (5.69, 9.46)	7.89	0.000	9	688	1121	23.3 %	0.236	1.000	0.843
	140 mg Q2W	6.90 (5.37, 8.43)	8.84	0.000	6	586	993	0.0 %	0.551	0.133	0.030
Non-HDL-C	420 mg Q4W	−52.11 (−55.07, −49.14)	34.42	0.000	8	672	1088	57.9 %	0.020	0.618	0.499
	140 mg Q2W	−56.07 (−61.67, −50.47)	19.62	0.000	6	586	993	89.0 %	0.000	0.452	0.616
TC/HDL-C	420 mg Q4W	−41.26 (−45.65, −36.87)	18.43	0.000	6	317	374	64.7 %	0.015	0.566	0.302
	140 mg Q2W	−44.85 (−49.11, −40.59)	20.64	0.000	4	229	285	63.6 %	0.041	0.089	0.126
VLDL-C	420 mg Q4W	−22.75 (−27.46, −18.04)	9.47	0.000	6	567	925	6.6 %	0.374	0.452	0.335
	140 mg Q2W	−24.83 (−38.29, −11.38)	3.62	0.000	4	480	831	82.3 %	0.001	0.734	0.462
ApoB	420 mg Q4W	−45.14 (−49.16, −41.12)	22.00	0.000	9	688	1121	78.8 %	0.000	0.076	0.027
	140 mg Q2W	−52.69 (−57.40, −47.98)	21.91	0.000	6	586	993	85.6 %	0.000	0.707	0.450
ApoA1	420 mg Q4W	5.17 (2.60, 7.73)	3.95	0.000	6	317	374	40.6 %	0.135	0.566	0.517
	140 mg Q2W	6.26 (1.71, 10.82)	2.69	0.007	4	230	285	74.5 %	0.008	0.308	0.129
ApoB/ApoA1	420 mg Q4W	−48.06 (−52.70, −43.43)	20.32	0.000	7	395	527	72.4 %	0.001	0.649	0.351
	140 mg Q2W	−53.68 (−57.77, −49.59)	25.74	0.000	5	305	438	65.8 %	0.020	0.806	0.500
TG	420 mg Q4W	−15.70 (−20.35, −11.05)	6.62	0.000	9	688	1121	42.5 %	0.084	0.118	0.030
	140 mg Q2W	−17.35 (−23.50, −11.20)	5.53	0.000	6	586	993	59.8 %	0.029	1.000	0.039
Lp(a)	420 mg Q4W	−25.40 (−29.09, −21.70)	13.47	0.000	9	688	1121	47.1 %	0.057	1.000	0.626
	140 mg Q2W	−32.39 (−38.92, −25.87)	9.73	0.000	6	586	993	79.3 %	0.000	1.000	0.819
PCSK9	420 mg Q4W	−44.04 (−53.90, −34.17)	8.75	0.000	6	540	908	85.2 %	0.000	0.452	0.473
	140 mg Q2W	−60.92 (−83.94, −37.89)	5.18	0.000	2	132	188	92.9 %	0.000	1.000	NA

ApoA1, apolipoprotein A1; ApoB, apolipoprotein B; ApoB/ApoA1, ratio of ApoB/ApoA1; CI, confidence interval; HDL-C, high-density lipoprotein (HDL) cholesterol; Lp(a), lipoprotein(a); NA, not applicable; Non-HDL-C, non-HDL cholesterol; PCSK9, proprotein convertase subtilisin/kexin type 9; TC, total cholesterol; TC/HDL-C, ratio of total cholesterol/HDL cholesterol; TG, triglycerides; VLDL-C, very low-density lipoprotein (VLDL) cholesterol

A significant increase in apolipoprotein A1 (ApoA1) was found at week 12 in all dosages of evolocumab except biweekly 105 mg administration. Monthly 420 mg and biweekly 140 mg treatment increased the ApoA1 level by 5.2 % (95 % CI: 2.6 to 7.7 %) and 6.3 % (95 % CI: 1.7 to 10.8 %) versus placebo, respectively (Table 5 and Additional file 2: Figure S6 and Table S11).

All dosages of evolocumab significantly lowered apolipoprotein B (ApoB), ApoB/ApoA1, and lipoprotein(a) (Lp(a)) at week 12, with monthly 420 mg treatment reducing levels by −45.1 % (95 % CI: −49.2 to −41.1 %), −48.1 % (95 % CI: −52.7 to −43.4 %), and −25.4 % (95 % CI: −29.1 to −21.7 %), respectively, versus placebo (Table 5 and Additional file 2: Figure S7 to S9, Tables S12 to S14). Modest to high levels of heterogeneity were found in both comparisons (*I*^*2*^ = 78.9 %, 72.4 %, and 47.1 %, respectively).

A significant decrease in triglycerides (TG) was found at week 12 in all dosages of evolocumab except biweekly 105 mg administration. Monthly 420 mg and biweekly 140 mg treatments lowered the TG level by −15.7 % (95 % CI: −20.4 to −11.1 %) and −17.4 % (95 % CI: −23.5 to −11.2 %) versus placebo, respectively (Table 5 and Additional file 2: Figure S10 and Table S15). A modest level of heterogeneity was detected.

The free PCSK9 level was decreased by any dosage of evolocumab treatment. At week 12, monthly 420 mg and biweekly 140 mg treatments lowered the PCSK9 level by −44.0 % (95 % CI: −53.9 to −34.2 %) and −60.9 % (95 % CI: −83.9 to −37.9 %) versus placebo, respectively (Table 5 and Additional file 2: Table S16). Significant heterogeneity was detected.

Similar results were obtained at the mean of weeks 10 and 12, and largely similar but less remarkable results were achieved when compared with ezetimibe (Additional file 2). Two RCTs reported efficacy outcomes of monthly 420 mg treatment at 52 weeks follow-up. Likewise, all the comparisons were significant (Additional file 2: Figure S11).

### Efficacy outcomes of alirocumab

**Fig. 5:**
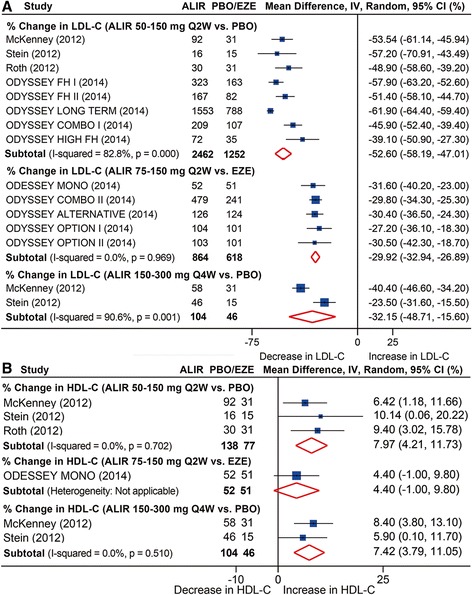
Pooled analysis for percent changes in LDL-C (**a**) and HDL-C (**b**) following alirocumab treatments stratified by dosages versus placebo or ezetimibe. ALIR, alirocumab; EZE, ezetimibe; PBO, placebo. HDL-C, high-density lipoprotein cholesterol; LDL-C, low density lipoprotein cholesterol

Both monthly and biweekly administration of alirocumab significantly lowered LDL-C levels, with biweekly 50 to 150 mg treatment reduced by over −50 % (mean reduction: −52.6 %, 95 % CI: −58.2 to −47.0 %) versus placebo, a less marked reduction was achieved when compared with ezetimibe (mean reduction: −29.9 %, 95 % CI: −32.9 to −26.9 %) and by monthly 150 to 300 mg treatment versus placebo (mean reduction: −32.2 %, 95 % CI: −48.7 to −15.6 %). Significant heterogeneity was detected in comparisons with placebo (Fig. 5A).

HDL-C level was increased by 8.0 % (95 % CI: 4.2 to 11.7 %) following biweekly 50 to 150 mg treatment and by 7.4 % (95 % CI: 3.8 to 11.1 %) after monthly 150 to 300 mg administration. No significant heterogeneity was found (*I*^*2*^ = 0 for both comparisons) (Fig. 5B). Meta-analyses of other efficiency outcomes demonstrated reduction in TC, non-HDL-C, ApoB, and Lp(a), and an increase in ApoA1 following alirocumab treatment, which are shown in Table S17 (in Additional file 2).

No significant publication bias was found in most analyses, detailed in each table in Additional file 2. Sensitivity analyses did not generated inconsistent results.

## Discussion

For the first time we provide in our study the rates of common adverse events following PCSK-9 antibody treatments by enrolling the largest sample size of patients and thus offering the most robust power, and detected largely no significant difference in major adverse events rates between antibody administration and control treatment, and no difference between different dosages of evolocumab. Notably, evolocumab reduced the rate of abnormal liver function, and alirocumab was associated with reduced rates of death and abnormal kidney function. Meanwhile, we determined the extent of LDL-C lowering of anti-PCSK9 antibodies: LDL-C level was reduced by over 50 % even though patients were on stable statin treatment. The extents of other favorable lipids changes were also documented in our meta-analyses.

It is worth noting that the favorable effects of anti-PCSK9 antibodies were largely achieved in populations who were already on stable statin treatments, indicating an additive, or even synergistic effect to statin in lowering LDL-C levels. This is not a surprise because statin therapy has been well documented to increase PCSK9 levels [40]; thus, inhibiting PCSK9 might enhance the LDL-C lowering effects of statins. Indeed, missense mutations in *PCSK9* increased response to statin therapy in unrelated hypocholesterolemic subjects and familial hypercholesterolemia patients [41]. Likewise, in our meta-analysis, the combination of anti-PCSK9 antibody and statin resulted in a very high-intensity LDL-lowering effect, which is recommended by the 2013 American College of Cardiology (ACC)/AHA guideline suggesting no LDL-C goals. Meta-analyses of RCTs on statins also demonstrated that further reductions in LDL-C produce definite further reductions in CVD events [42], even in people at low risk of CVDs [43].

Two fundamental elements could lead to safety concerns: firstly those due to monoclonal antibody administration and secondly due to achieving very low LDL-C levels. We were unable to obtain the mean LDL-C level at the end of antibody administration due to lack of patient-level data in our study. However, estimated lowest LDL-C levels from observations of each study were less than 50 mg/dl, which was more remarkable than those achieved in the recently presented IMPROVE-IT trial (Improved reduction of outcomes: vytorin efficacy international trial) [44]. A combination of simvastatin and ezetimibe led to a mean LDL-C level as low as 53.2 mg/dl at one-year follow-up in high-risk patients with acute coronary syndrome, but showed good safety outcomes, indicating that an even lower level of LDL-C might not result in safety concerns. This notion was further confirmed by two recently published trials with regard to both evolocumab and alirocumab, with a longer follow-up of approximately 12 months [23, 45]. The OSLER trial, an extension trial of several phase 2 and phase 3 parent trials (most of which were included in our meta-analysis), showed similar rates of adverse events in patients with LDL-C levels less than 40 mg/dl or less than 25 mg/dl as in those with higher LDL-C levels following evolocumab treatment [45]. The full-term follow-up of the ODYSSEY LONG TERM trial also revealed similar frequency of adverse events among patients who had a LDL-C level less than 25 mg/dl and those who did not [23]. More straightforwardly in our meta-analysis, administration of both anti-PCSK9 antibodies showed promising safety profiles, except that administration of alirocumab was associated with a higher rate of injection-site reactions.

Whether anti-PCSK9 antibody treatments could translate into improved cardiovascular outcomes remains to be confirmed. The ongoing FOURIER (NCT01764633) and ODYSSEY OUTCOMES (NCT01663402) trials will answer this question by assessing the effect of evolocumab and alirocumab on major CVD events with about five years follow-up. However, the probable clinical benefits could be preliminarily inferred based on current evidence: 1) anti-PCSK9 antibodies substantially reduced LDL-C, non-HDL-C and ApoB levels, all of which are positively associated with CVD events [46], and ‘a lower LDL-C, a better outcome’ has been indicated not only in the era of statins but also following combined use of statins and ezetimibe [44]; 2) anti-PCSK9 antibodies significantly increased HDL-C and ApoA1 levels, which are strongly associated with reduced CVD risk, even in patients achieving very low LDL-C [47]; 3) in the ARIC study, loss-of-function PCSK9 mutations resulted in 28 % (15 %) reduction in LDL-C, and 88 % (47 %) reduction in CHD risk in African-Americans (white people) [48]; combined analyses in other cohort studies also generated 30 % reduction in ischemic heart disease risk [49]; and 4) more direct evidence from the longer-term follow-up results of the OSLER and ODYSSEY LONG TERM trials, although with a limitation of exploratory analysis, both of these trials suggested that patients receiving anti-PCSK9 antibodies had a significantly lower risk of major adverse cardiovascular events, which is consistent with our study showing alirocumab reduced the rates of death. Notably, both trials demonstrated that the cumulative incidence curves diverged progressively over time; therefore, a more remarkable benefit might be expected given a longer-term follow-up. Provided the exploratory nature of these trials, the limited follow-up length and the small number of cardiovascular events, results from ongoing FOURIER (over 27,500 high-risk patients with cardiovascular disease) and ODYSSEY OUTCOMES trials (over 18,000 patients who have experienced an acute coronary syndrome event 4 to 52 weeks prior to randomization) are urgently needed to provide definite answers.

### Study limitations

First, the meta-analysis was based on study-level instead of patient-level data. Second, a high level of heterogeneity exists in several analyses. Heterogeneities in patient profile (unrelated or familial hypercholesterolemia) and background lipid-lowering therapy (maximum-tolerated statin, statin-intolerance, or no background anti-lipid therapy) are likely to account for part of this heterogeneity. We performed subgroup analyses based on the type of study population and heterogeneity still existed (data not show). Therefore, we pooled these data with random-effects models. Third, additional ongoing trials evaluating the efficacy and safety of alirocumab are to be published in a few years. However, with respect to primary efficacy endpoint, dramatic upregulating-LDL effects needed to be reported to balance the lowering-LDL effects demonstrated in our study given the number of patients known to have participated in these ongoing trials (ODYSSEY CHOICE I, ODYSSEY OLE, and so on), which is unlikely. Fourth, with respect to analysis on safety profiles, wide-range 95 % CIs were observed in several endpoints, which made precise estimation of the incidences of these endpoints impossible. Meanwhile, several composite endpoints were included in our study, such as adjudicated cardiovascular events, which might lower the ability of detecting each individual endpoint. Fifth, most trials included in our study had a relatively short-term follow-up (12 and 52 weeks for evolocumab, and mostly 24 weeks for alirocumab), thus rare events could not be fully revealed. Sixth, we could not rule out bias of selective reporting on several safety outcomes; to minimize this bias, we reviewed all the materials (including supplementary materials and relevant publications in other papers) provided by these studies and extracted and analyzed all these data. Notably, no obvious selective reporting bias was detected in major safety endpoints, such as any TEAEs, serious TEAEs, abnormal liver function, abnormal kidney function, injection-site reactions, musculoskeletal disorders and so on. Seventh, most patients enrolled are white; therefore, caution should be taken to interpret in other populations.

## Conclusions

Evolocumab and alirocumab were safe and well-tolerated, largely showing no significant differences in rates of common adverse events with placebo or ezetimibe controls. No difference was detected following different dosages of evolocumab treatments regarding safety profiles. Both anti-PCSK9 antibodies substantially reduced LDL-C by over 50 %, increased HDL-C levels, and resulted in favorable changes in other lipids. We await the results of ongoing trials evaluating their effects on CVD events.

## Additional files

Additional file 1:
**PRISMA 2009 Checklist.**
Additional file 2: Table S1. Additional baseline characteristics of included randomized trials. **Table S2.** Safety endpoints and monitoring methods (mainly visiting periods) of included randomized trials. **Table S3.** Risk of bias analysis for included randomized trials. **Figure S1.** Forest plot demonstrating absolute changes in LDL cholesterol (LDL-C) stratified by dosages following evolocumab treatments versus placebo at 12 weeks follow-up. **Table S4.** Percent and absolute changes of LDL cholesterol (LDL-C) at week 12 after evolocumab treatment versus placebo or ezetimibe. **Table S5.** Percent and absolute changes of LDL cholesterol at mean of weeks 10 and 12 after evolocumab treatment versus placebo or ezetimibe. **Table S6 to S15.** Percent change of HDL cholesterol (**Table S6**), total cholesterol (**Table S7**), total cholesterol/HDL-C ratio (**Table S8**), non-HDL cholesterol (**Table S9**), VLDL cholesterol (VLDL-C, **Table S10**), apolipoprotein A1 (ApoA1, **Table S11**), apolipoprotein B (ApoB, **Table S12**), ApoB/ApoA1 ratio (**Table S13**), lipoprotein(a) (Lp(a), **Table S14**), and triglycerides (TG, **Table S15**) at week 12 and at mean of weeks 10 and 12 after evolocumab treatment versus placebo or ezetimibe. **Figure S2 to S10.** Forest plot demonstrating changes in total cholesterol (TC, **Figure S2**), total cholesterol/HDL-C ratio (**Figure S3**), non-HDL cholesterol (non-HDL-C, **Figure S4**), VLDL cholesterol (VLDL-C, **Figure S5**), apolipoprotein A1 (ApoA1, **Figure S6**), apolipoprotein B (ApoB, **Figure S7**), ApoB/ApoA1 ratio (**Figure S8**), lipoprotein(a) (Lp(a), **Figure S9**), and triglycerides (TG, **Figure S10**) stratified by dosages following evolocumab treatments versus placebo at 12 weeks follow-up. **Table S16.** Percent change of PCSK9 at week 12 after evolocumab treatment versus placebo. **Figure S11.** Forest plot demonstrating changes in lipid profiles following monthly 420 mg evolocumab treatments versus placebo at 52 weeks follow-up. **Table S17.** Percent changes of other endpoints following alirocumab treatment versus placebo or ezetimibe.

## Abbreviations

ADH: autosomal dominant hypercholesterolemia
AST/ALT: aspartate aminotransferase/alanine aminotransferase
CHD: coronary heart disease
CI: confidence interval
CK: creatine kinase
CVD: cardiovascular disease
HDL-C: high-density lipoprotein cholesterol
LDL-C: low-density lipoprotein cholesterol
LDLR: LDL receptor
PCSK9: proprotein convertase subtilisin/kexin type 9
RCT: randomized controlled trial
RR: relative risk
TC: total cholesterol
TEAE: treatment emergent adverse event
TG: triglycerides
ULN: upper limit of normal
VLDL-C: very low-density lipoprotein cholesterol
