# Supplementary material for: Safety and efficacy of anti-PCSK9 antibodies: a meta-analysis of 25 randomized, controlled trials
Source: BMC Med. 2015 Jun 23;13:123. doi: 10.1186/s12916-015-0358-8 (PMC4477483; doi:10.1186/s12916-015-0358-8)
Supplement: Additional file 2: Table S1. — Additional baseline characteristics of included randomized trials. Table S2. Safety endpoints and monitoring methods (mainly visiting periods) of included randomized trials. Table S3. Risk of bias analysis for included randomized trials. Figure S1. Forest plot demonstrating absolute changes in LDL cholesterol (LDL-C) stratified by dosages following evolocumab treatments versus placebo at 12 weeks follow-up. Table S4. Percent and absolute changes of LDL cholesterol (LDL-C) at week 12 after evolocumab treatment versus placebo or ezetimibe. Table S5. Percent and absolute changes of LDL cholesterol at mean of weeks 10 and 12 after evolocumab treatment versus placebo or ezetimibe. Table S6 to S15. Percent change of HDL cholesterol (Table S6), total cholesterol (Table S7), total cholesterol/HDL-C ratio (Table S8), non-HDL cholesterol (Table S9), VLDL cholesterol (VLDL-C, Table S10), apolipoprotein A1 (ApoA1, Table S11), apolipoprotein B (ApoB, Table S12), ApoB/ApoA1 ratio (Table S13), lipoprotein(a) (Lp(a), Table S14), and triglycerides (TG, Table S15) at week 12 and at mean of weeks 10 and 12 after evolocumab treatment versus placebo or ezetimibe. Figure S2 to S10. Forest plot demonstrating changes in total cholesterol (TC, Figure S2), total cholesterol/HDL-C ratio (Figure S3), non-HDL cholesterol (non-HDL-C, Figure S4), VLDL cholesterol (VLDL-C, Figure S5), apolipoprotein A1 (ApoA1, Figure S6), apolipoprotein B (ApoB, Figure S7), ApoB/ApoA1 ratio (Figure S8), lipoprotein(a) (Lp(a), Figure S9), and triglycerides (TG, Figure S10) stratified by dosages following evolocumab treatments versus placebo at 12 weeks follow-up. Table S16. Percent change of PCSK9 at week 12 after evolocumab treatment versus placebo. Figure S11. Forest plot demonstrating changes in lipid profiles following monthly 420 mg evolocumab treatments versus placebo at 52 weeks follow-up. Table S17. Percent changes of other endpoints following alirocumab treatment versus placebo or ezetimibe. [file 12916_2015_358_MOESM2_ESM.doc]

**Additional Files 2**

**Safety and Efficacy of Anti-PCSK9 Antibodies: A Meta-analysis of 25 Randomized, Controlled Trials**

Xin-Lin Zhang^1,*^, Qing-Qing Zhu^2,*^, Li Zhu ^3,*^, Jian-Zhou Chen^1^, Qin-Hua Chen^1^, Guan-Nan Li^1^, Jun Xie^1^, Li-Na Kang^1^, Biao Xu^1,§^

^1^Department of Cardiology, Affiliated Drum Tower Hospital, Nanjing University School of Medicine, Nanjing, China

^2^Department of Respiratory Medicine, Jinling Hospital, Nanjing University School of Medicine, Nanjing, China

^3^Department of Radiology, Affiliated Drum Tower Hospital, Nanjing University School of Medicine, Nanjing, China

^*^These authors contributed equally to this work.

^§^Corresponding author: Dr. Biao Xu, Department of Cardiology, Affiliated Drum Tower Hospital, Nanjing University School of Medicine, 321 Zhongshan Road, 210008 Nanjing, Jiangsu Province, China. Tel: +86 25 831 052 05; fax: +86 25 833 080 59. Email: [xubiao@medmail.com.cn](mailto:xubiao@medmail.com.cn).

**Table S1. Additional baseline characteristics of included randomized trials.**

| **Study** | **Year** | **White, n (%)** | **Black, n (%)** | **Other, n (%)** | **Non-HDL-C, mmol/L** | **Total-C/HDL-C ratio** | **VLDL-C, mmol/L** | **ApoB, g/L** | **ApoA1, g/L** | **Apo B/Apo A1 ratio** | **TG, mmol/L** | **Lp(a), nmol/L** | **Treatments and doses** |
| --- | --- | --- | --- | --- | --- | --- | --- | --- | --- | --- | --- | --- | --- |
| RUTHERFORD | 2012 | 148 (88) | 4 (3) | 15 (9) | 4.7 (1.4) | 4.9 (1.8) | 0.6 (0.4) | 1.3 (0.3) | 1.5 (2.8) | 0.9 (0.3) | 1.4 (0.8) | 45.0 (12.5, 154.5) | Evolocumab 350 mg, 420 mg, or placebo Q4W |
| LAPLACE-TIMI 57 | 2012 | 560 (89) | 50 (8) | 21 (3) | 3.8 (0.8) | 4.0 (1.2) | 0.6 (0.3) | 1.0 (0.2) | 1.6 (0.3) | 0.7 (0.2) | 1.5 (0.7) | 43.0 (13.0, 161.0) | Evolocumab 70 mg, 105 mg, 140 mg, or placebo Q2W OR Evolocumab 280 mg, 350 mg, 420 mg, or placebo Q4W |
| GAUSS | 2012 | 139 (88) | 8 (5) | 10 (7) | 5.8 (1.4) | 5.4 (2.3) | 0.8 (0.4) | 1.4 (0.4) | 1.6 (0.3) | 0.9 (0.3) | 1.9 (0.8) | 39.0 (11.0, 137.0) | Evolocumab 280 mg, Evolocumab 280 mg, 350 mg, or 420 mg Q4W OR ezetimibe 10 mg and placebo Q4W OR ezetimibe 10 mg and Evolocumab 420 mg Q4W |
| MENDEL | 2012 | 319 (79) | 64 (16) | 23 (6) | 4.3 (0.7) | 4.51 (1.35) | 0.7 (0.4) | 1.1 (0.2) | 1.5 (0.3) | 0.7 (0.2) | 1.5 (0.8) | 71.2 (76.1) | Evolocumab 70 mg, 105 mg, or 140 mg or placebo Q2W OR Evolocumab 280 mg, 350 mg, or 420 mg or placebo Q4W OR Daily ezetimibe 10 mg |
| YUKAWA | 2014 | 0 | 0 | 307 (100) | 4.3 (0.6) | 4.3 (1.0) | 0.6 (0.5, 0.9) | 1.1 (0.2) | 1.6 (0.3) | 0.7 (0.2) | 1.5 (0.6) | 33.5 (13.0, 66.0) | Placebo, Evolocumab 70 mg, or Evolocumab 140 mg Q2 W; or placebo, Evolocumab 280 mg, or Evolocumab 420 mg Q4W |
| MENDEL-2 | 2014 | 510 (83) | 40 (7) | 64 (10) | 4.3 (0.7) | 4.0 (1.0) | 0.6 (0.4, 0.9) | 1.0 (0.2) | 1.6 (0.4) | 0.7 (0.2) | 1.3 (0.9, 2.0) | 21.0 (9.0, 49.0) | Placebo PO and placebo SC Q2W, or placebo PO and placebo SC Q4W, or ezetimibe PO and placebo SC Q2W, or ezetimibe PO and placebo SC Q4W, or placebo PO and Evolocumab 140 mg Q2W, or placebo PO and Evolocumab 420 mg Q4W |
| LAPLACE-2 | 2014 | 1782 (94) | 75 (4) | 39 (2) | 3.5 (1.1) | NA | 0.6 (0.4, 0.8) | 0.9 (0.3) | NA | NA | 1.3 (1.0, 1.6) | 34.0 (12.0, 149.0) | Evolocumab (SC, 140 mg Q2W) or placebo (SC, Q2W), or Evolocumab (420 mg, SC, Q4W) or placebo (SC, Q4W). Evolocumab (140 mg, SC, Q2W) and placebo (PO, QD), Evolocumab (420 mg, SC, Q4W) and placebo (PO, QD), placebo (SC, Q2W) and placebo (PO, QD) or ezetimibe (10 mg, PO, QD), or placebo (SC, Q4W) and placebo (PO, QD) or ezetimibe (10 mg, PO, QD) |
| GAUSS-2 | 2014 | 287 (94) | 7 (2) | 13 (4) | 6.0 (1.7) | 6.0 (2.0) | 0.9 (0.6, 1.3) | 1.4 (0.4) | 1.5 (0.2) | 0.9 (0.3) | 1.9 (1.4, 2.7) | 57.0 (22.0, 205.0) | Ezetimibe QD and placebo Q2W, or Evolocumab 140mg Q2W and placebo QD, or ezetimibe QD and placebo Q4W, or Evolocumab 420mg Q4W and placebo QD |
| DESCARTES | 2014 | 724 (80) | 76 (8) | 101 (12) | 3.3 (0.7) | 3.6 (1.1) | 0.6 (0.3) | 0.9 (0.2) | 1.6 (0.3) | 0.6 (0.2) | 1.2 (1.0, 1.7) | 67 (24.0,180.0) | Placebo or Evolovumab 420 mg Q4W in background groups (diet alone), atorvastatin 10 mg QD, atorvastatin 89 mg QD, and atorvastatin 80 mg plus ezetimibe 10 mg QD) |
| OSLER | 2014 | 972 (88) | 79 (7) | 53 (5) | 4.4 (1.2) | 4.6 (1.7) | 0.6 (0.4, 0.8) | 1.1 (0.3) | 1.5 (0.3) | 0.8 (0.2) | 1.4 (1.0, 1.9) | 36 (11.0, 115.0) | Evolocumab 420 mg SC Q4W and SOC, or SOC alone |
| TESLA | 2014 | 44 (90) | NA | NA | NA | NA | NA | 2.1 (0.7) | 1.1 (0.3) | NA | 1.2 (0.6) | 101.0 (31.0, 146.0) | Evolocumab 420 mg or placebo Q4W |
| RUTHERFORD-2 | 2014 | 296 (90) | NA | NA | NA | NA | NA | 1.1 (0.3) | 1.5 (0.3) | NA | 1.1 (0.8, 1.6) | 44.0 (24.0, 105.0) | Evolocumab 140 mg SC Q2W, or Evolocumab 420 mg SC Q4W, or placebo SC Q2W, or placebo SC Q4W |
| McKenney | 2012 | 158 (86.3) | 23 (13) | 42 (23) | 4.1 (0.7) | NA | NA | 1.1 (0.2) | 1.4 (1.3, 1.6) | NA | 1.4 (1.0, 2.1) | 50 (25, 225) | Placebo Q2W, or Alirocumab 50, 100, or 150 mg Q2W, or Alirocumab 200 or 300 mg Q4W alternating with placebo to mimic Q2W dosing |
| Stein | 2012 | 73 (95) | NA | NA | 4.7 (1.0) | NA | NA | 1.3 (0.3) | 1.6 (0.2) | NA | 1.4 (0.9, 2.5) | 72.5 (12.5, 320) | Alirocumab 150 mg, 200 mg, or 300 mg Q4W, or 150 mg Q2W, or placebo Q2W |
| Roth | 2012 | 80 (87) | 12 (13) | 0 | 3.8 (0.6) | NA | NA | 1.0 (0.2) | 1.6 (0.3) | NA | 1.3 (1.0, 1.8) | 57.5 (15, 140) | Atorvastatin 80 mg and placebo, or atorvastatin 10 mg and Alirocumab 150mg Q2W, or atorvastatin 80 mg and Alirocumab 150mg Q2W |
| ODYSSEY COMBO II | 2014 | 610 (85) | NA | NA | NA | NA | NA | NA | NA | NA | NA | NA | Ezetimibe 10 mg PO QD and placebo Q2W SC, or Alirocumab 75-150 mg Q2W SC and placebo PO QD |
| ODYSSEY FH I | 2014 | 444 (91) | NA | NA | NA | NA | NA | NA | NA | NA | NA | NA | Alirocumab 75-150 mg or placebo Q2W |
| ODYSSEY FH II | 2014 | 244 (98) | NA | NA | NA | NA | NA | NA | NA | NA | NA | NA | Alirocumab 75-150 mg or placebo Q2W |
| ODYSSEY LONG TERM | 2014 | 2171 (93) | NA | NA | NA | NA | NA | NA | NA | NA | NA | NA | Alirocumab 150 mg or placebo Q2W |
| ODESSEY MONO | 2014 | 93 (90) | 10 (10) | 0 | 4.2 (0.8) | NA | NA | 1.0 (0.2) | 1.6 (0.3) | NA | 1.3 (1.0, 1.7) | 40 (15, 85) | Ezetimibe 10 mg PO QD and placebo Q2W SC, or Alirocumab 75-150 mg Q2W SC and placebo PO QD |
| ODYSSEY ALTERNATIVE | 2014 | 233 (93) | NA | NA | 5.9 (2.1) | NA | NA | 1.4 (0.4) | 1.5 (0.2) | NA | 1.6 (1.1, 2.5) | 135 (17.5, 107.5) | Ezetimibe 10 mg PO QD and placebo Q2W SC, or Alirocumab 75-150 mg Q2W SC and placebo PO QD |
| ODYSSEY COMBO I | 2014 | 258 (82) | NA | NA | 3.4 (1.0) | NA | NA | 0.9 (0.2) | NA | NA | 1.4 (1.1, 2.0) | 95 (25, 175) | Alirocumab 75-150 mg or placebo Q2W |
| ODYSSEY HIGH FH | 2014 | 94 (88) | NA | NA | 6.0 (1.2) | NA | NA | 1.5 (0.3) | NA | NA | NA | 75 (27.5, 105) | Alirocumab 75-150 mg or placebo Q2W |
| ODYSSEY OPTION I | 2014 | 182 (89) | NA | NA | NA | NA | NA | NA | NA | NA | NA | NA | Alirocumab 75-150 mg Q2W + ATV 20/40 mg; EZE + ATV 20/40 mg |
| ODYSSEY OPTION II | 2014 | 175 (86) | NA | NA | NA | NA | NA | NA | NA | NA | NA | NA | Alirocumab 75-150 mg Q2W + RSV 20/40 mg; EZE + RSV 20/40 mg |

Data are mean (SD), mean (SE), n (%), or median (IQR). Lipid profiles are mean (SE) if not indicated.

ApoA1 = apolipoprotein A1; ApoB = apolipoprotein B; ApoB/ApoA1 = ratio of ApoB/ApoA1; Lp(a) = lipoprotein(a); NA = not applicable; Non-HDL-C = Non-HDL cholesterol; TC/HDL-C = ratio of total cholesterol/HDL cholesterol; TG = triglycerides; VLDL-C = very low-density lipoprotein (VLDL) cholesterol; NA = not applicable; PO = per os; QD = quāque diē; Q2W = every two weeks; Q4W = every four weeks; SC = subcutaneously; SOC = standard-of-care.

DESCARTES = the Durable Effect of PCSK9 Antibody Compared with Placebo Study trial; GAUSS = the Goal Achievement after Utilizing an anti-PCSK9 antibody in Statin Intolerant Subjects trial; LAPLACE-TIMI 57 = the LDL-C Assessment With PCSK9 Monoclonal Antibody Inhibition Combined With Statin Therapy (LAPLACE)–Thrombolysis in Myocardial Infarction (TIMI) 57 trial; MENDEL = Monoclonal Antibody Against PCSK9 to Reduce Elevated LDL-C in Subjects Currently Not Receiving Drug Therapy for Easing Lipid Levels trial; ODYSSEY COMBO II = Efficacy and Safety of Alirocumab Versus Ezetimibe on Top of Statin in High Cardiovascular Risk Patients With Hypercholesterolemia; ODYSSEY FH I/II = Efficacy and Safety of Alirocumab in Patients With Heterozygous Familial Hypercholesterolemia Not Adequately Controlled With Their Lipid-Modifying Therapy; ODESSEY LONG TERM = Long-term Safety and Tolerability of Alirocumab Versus Placebo on Top of Lipid-Modifying Therapy in High Cardiovascular Risk Patients With Hypercholesterolemia; ODESSEY MONO = Monotherapy with alirocumab versus ezetimibe in patients with hypercholesterolemia; OSLER = the Open Label Study of Long Term Evaluation Against LDL-C trial; RUTHERFORD = The Reduction of LDL-C With PCSK9 Inhibition in Heterozygous Familial Hypercholesterolemia Disorder trial; TESLA = The Trial Evaluating PCSK9 Antibody in Subjects with LDL Receptor Abnormalities; YUKAWA = the StudY of LDL-Cholesterol Reduction Using a Monoclonal PCSK9 Antibody in Japanese Patients With Advanced Cardiovascular Risk trial.

**Table S2. Safety endpoints and monitoring methods (mainly visiting periods) of included randomized trials.**

| **Trial/first author** | **Year** | **Safety endpoints** | **Monitoring methods** |
| --- | --- | --- | --- |
| RUTHERFORD | 2012 | Safety endpoints included treatment-emergent and serious adverse events, creatine kinase (CK) and hepatic enzyme elevations, and anti-evolocumab antibodies. | Study visits were at weeks 1, 0 (day 1), 2, 4, 8, and 12. At each visit, patients were required to be fasting; blood was drawn for lipids and other laboratory measurements, vital signs were measured, patients were questioned for adverse events and concomitant medication, stable diet was encouraged, and an ECG was performed and read centrally. |
| LAPLACE-TIMI 57 | 2012 | Key safety end points included the frequency of treatment-emergent adverse events, adjudicated myalgia, laboratory values, vital signs, electrocardiographic parameters, and formation of anti-AMG 145 antibody | During in-person follow-up visits every 2 weeks, fasting laboratory measurements, adverse events, clinical endpoints, and concomitant treatments were assessed in all patients. |
| GAUSS | 2012 | Key safety end points included the incidence of treatment-emergent adverse events (AEs), laboratory values, vital signs, and electrocardiographic parameters at each scheduled visit, and the incidence of anti-AMG145 antibodies (binding and neutralizing). | The treatment period was 12 weeks, with study visits scheduled at screening and day 1 and at weeks 2, 4, 8, and 12. Blood samples for all assessments were collected after an overnight fast (water only) and analyzed by a central laboratory. |
| MENDEL | 2012 | Safety endpoints included frequency of treatment-emergent adverse events, anti-AMG 145 antibodies, and muscle-related events. | Study visits were scheduled at 2 week intervals until the end of the study at week 12 (every 4 week groups) or week 14 (every 2 week groups) |
| YUKAWA | 2014 | Safety endpoints included the incidence of adverse events (AEs), laboratory values and vital signs, electrocardiography (ECG) parameters, and incidence of anti-evolocumab antibodies. | Study visits were at screening, and at weeks 0 (day 1), 2, 4, 6, 8, 10, and 12. |
| MENDEL-2 | 2014 | Key safety endpoints included the incidence of treatment-emergent AEs, serious AEs, development of anti-evolocumab antibodies, and increases of hepatic enzymes 3 times, bilirubin 2 times, and creatine kinase 5 times above the upper limit of normal. | Study visits were at screening, and at weeks 0 (day 1), 2, 4, 6, 8, 10, and 12. |
| LAPLACE-2 | 2014 | Safety endpoints included incidence of adverse events, serious adverse events, and anti-evolocumab antibodies. Safety laboratory studies included measurement of transaminase, bilirubin, and creatine kinase levels. | Study visits were at screening, and at weeks 0 (day 1), 2, 4, 6, 8, 10, 12 and 14. |
| GAUSS-2 | 2014 | Safety endpoints included treatment-emergent and serious adverse events, creatine kinase (CK) and hepatic enzyme elevations, and anti-evolocumab antibodies. | Study visits were at screening, and at weeks 0 (day 1), 2, 4, 6, 8, 10, and 12. |
| DESCARTES | 2014 | Long-term safety and side-effect profiles were assessed by means of adverse-event reporting, clinical examination, and laboratory testing. | Study visits were scheduled every 4 weeks, with additional visits at weeks 13 and 37. |
| OSLER | 2014 | Safety end points included the incidence of AEs, serious AEs, and AEs leading to discontinuation of investigational product. Other safety end points included the incidence of creatine kinase and liver function test abnormalities and the incidence and percentage of patients who developed anti-evolocumab antibodies (binding or neutralizing). | Study visits occurred every 4 weeks for patients randomized to evolocumab+SOC. After their week 4 visits, patients randomized to SOC returned to the study center only for quarterly visits thereafter (weeks 12, 24, 36, 48, and 52); all other interval visits (every 4 weeks) were conducted over the phone. Sites collected blood samples at week 4 and at the quarterly visits. |
| TESLA | 2014 | The main safety endpoints were treatment-emergent and serious adverse events, safety laboratory assessments, electro cardiograph parameters, and the development of anti-evolocumab antibodies. | Study visits took place at the screening visit, at randomization (day 1), and at weeks 4, 6, 8, and 12, with optional visits at weeks 2 and 10. |
| RUTHERFORD-2 | 2014 | Key safety outcomes included treatment-emergent and serious adverse events, increases in creatine kinase and hepatic enzymes, the development of anti-evolocumab anti bodies, and adjudicated cardiovascular adverse events. | Study visits were at screening, and at weeks 0 (day 1), 2, 8, 10, and 12. |
| McKenney | 2012 | Safety was assessed throughout the study by clinical examination, vital signs, AEs, serious AEs, laboratory tests, and 12-lead electrocardiogram. AE data were collected from screening onwards. | Visits during the treatment period were every 2 weeks. |
| Stein | 2012 | Clinical examination, including supine systolic/diastolic blood pressure and adverse event, electrocardiographs, laboratory tests, including alanine aminotransferase, aspartate aminotransferase, high-sensitivity C-reactive protein, and creatine phosphokinase | Safety was assessed at every visit. Electrocardiographs were  done at weeks 0, 6, and 12. |
| Roth | 2012 | Safety was assessed by means of clinical examination, reporting of adverse events, 12-lead electrocardiography, measurement of vital signs, and laboratory testing that included hematologic tests, liver-function tests, and measurement of creatine kinase levels. | Anti-SAR236553 antibodies were assayed at baseline, week 4, and week 8, as well as at week 12 and week 16 (follow-up visits). Data on adverse events were collected from the time the patient signed the consent form until the end of the study. |
| ODYSSEY COMBO II | 2014 | Safety was assessed by analyzing AEs (including adjudicated cardiovascular events), laboratory parameters, and vital signs | On-site patient assessments were scheduled at regular intervals from randomization to week 104 (end of treatment visit): at randomization and then weeks 4, 8, 12, 16, 24, 36, 52, 64, 76, 88 and 104. |
| ODYSSEY FH I, II and HIGH FH [1] | 2014 | Safety parameters (AEs, laboratory data [blood biochemistry, hematology and urinalysis], vital signs and electrocardiogram) | On-site patient assessments take place at Weeks 0, 12, 24, 36, 52, 64, 78, and 86, the end-of-study visit. |
| ODYSSEY LONG TERM | 2014 | Safety end points were adverse events, including symptoms, laboratory abnormalities, vital-sign abnormalities, electrocardiographic abnormalities, and adjudicated cardiovascular events | Patients were to return to the study site during the double-blind study period at weeks 0 (baseline), 4, 8, 12, 16, 24, 36, 52, 64, and 78, and again 8 weeks after the end of the double-blind period (i.e., at week 86) for a safety assessment. |
| ODYSSEY MONO | 2014 | Safety was assessed throughout the study by adverse event (AE) reporting, local tolerability (injection site reactions), laboratory data, vital signs, physical signs, and electrocardiogram. | Assessments over the double-blind period were carried out at weeks 0, 4, 8, 12, 16, and 24 |
| ODYSSEY ALTERNATIVE [2] | 2014 | General safety is being assessed as treatment-emergent AEs, blood biochemistry, hematology, urinalysis abnormalities, vital signs, electrocardiogram, and development of anti-alirocumab antibodies at the time points given previously for the lipid assessments. Injection site reactions will be closely monitored as AEs of special interest. | Assessed at 0, 4, 8, 12, 16, and 24 weeks of the double-blind period and every 4 weeks during the open-label extension up to week 36, then at week 52 and every 12 weeks thereafter |
| ODYSSEY COMBO I [3] | 2014 | Safety was assessed by analyzing AEs (including adjudicated cardiovascular events), laboratory parameters, and vital signs | On-site patient assessments during the treatment period were scheduled at randomization and then weeks 4, 8, 12, 16, 24, 36, and 52 (end of treatment visit) |
| ODYSSEY OPTION I and II [4] | 2014 | Safety events (AEs, injection-site reactions, laboratory data [blood biochemistry, hematology, and urinalysis], vital signs and electrocardiogram) | On-site patient assessments took place at randomization and then at weeks 4, 8, 12, 16, and 24, and at the end-of-study visit, week 32. |

References of several trials were presented here because these trials have not been published yet; all references presented were rations and designs of these trials.

AE = adverse events; CK = creatine kinase; ECG = electrocardiography. Other abbreviations as in Table S1.

**Table S3. Risk of bias analysis for included randomized trials.**

| **Study** | **Year** | **Random sequence generation (selection bias)** | **Allocation concealment (selection bias)** | **Blinding of participants and personnel (performance bias)** | **Blinding of outcome assessment (detection bias)** | **Incomplete outcome data (attrition bias)** | **Selective reporting (reporting bias)** | **Other bias*** |
| --- | --- | --- | --- | --- | --- | --- | --- | --- |
| RUTHERFORD | 2012 | Unclear | Unclear | Low | Low | Low | Low | Low |
| LAPLACE-TIMI 57 | 2012 | Low | Low | Low | Low | Low | Low | Low |
| GAUSS | 2012 | Unclear | Low | Low | Low | Low | Low | High |
| MENDEL | 2012 | Low | Low | Low | Low | Low | Low | High |
| YUKAWA | 2014 | Unclear | Unclear | Low | Unclear | Low | Low | Low |
| MENDEL-2 | 2014 | Low | Low | Low | Low | Low | Low | Unclear |
| LAPLACE-2 | 2014 | Unclear | Low | Low | Low | Low | Low | Unclear |
| GAUSS-2 | 2014 | Unclear | Unclear | Low | Low | Low | Low | Unclear |
| DESCARTES | 2014 | Low | Low | Low | Low | Low | Low | High |
| OSLER | 2014 | Unclear | Unclear | Low | Unclear | Low | Low | Low |
| TESLA | 2014 | Low | Low | Low | Low | Low | Low | Low |
| RUTHERFORD-2 | 2014 | Low | Low | Low | Low | Low | Low | Low |
| McKenney | 2012 | Unclear | Unclear | Low | Unclear | Low | Low | Low |
| Stein | 2012 | Low | Low | Low | Low | Low | Low | High |
| Roth | 2012 | Low | Low | Low | Low | Low | Low | Low |
| ODYSSEY COMBO II | 2014 | Unclear | Unclear | Low | Low | Low | Low | Unclear |
| ODYSSEY FH I | 2014 | Low | Low | Low | Low | Low | Low | Unclear |
| ODYSSEY FH II | 2014 | Low | Low | Low | Low | Low | Low | Unclear |
| ODYSSEY LONG TERM | 2014 | Unclear | Unclear | Low | Low | Low | Low | Unclear |
| ODESSEY MONO | 2014 | Unclear | Low | Low | Low | Low | Low | Unclear |
| ODYSSEY ALTERNATIVE | 2014 | Low | Low | Low | Low | Low | Low | Unclear |
| ODYSSEY COMBO I | 2014 | Unclear | Unclear | Low | Low | Low | Low | Unclear |
| ODYSSEY HIGH FH | 2014 | Low | Low | Low | Low | Low | Low | Unclear |
| ODYSSEY OPTION I | 2014 | Unclear | Unclear | Low | Low | Low | Low | Unclear |
| ODYSSEY OPTION II | 2014 | Unclear | Unclear | Low | Low | Low | Low | Unclear |

*The assessment of other bias was mainly based on the role of sponsors in data collection and management. Other abbreviations as in Table S1.


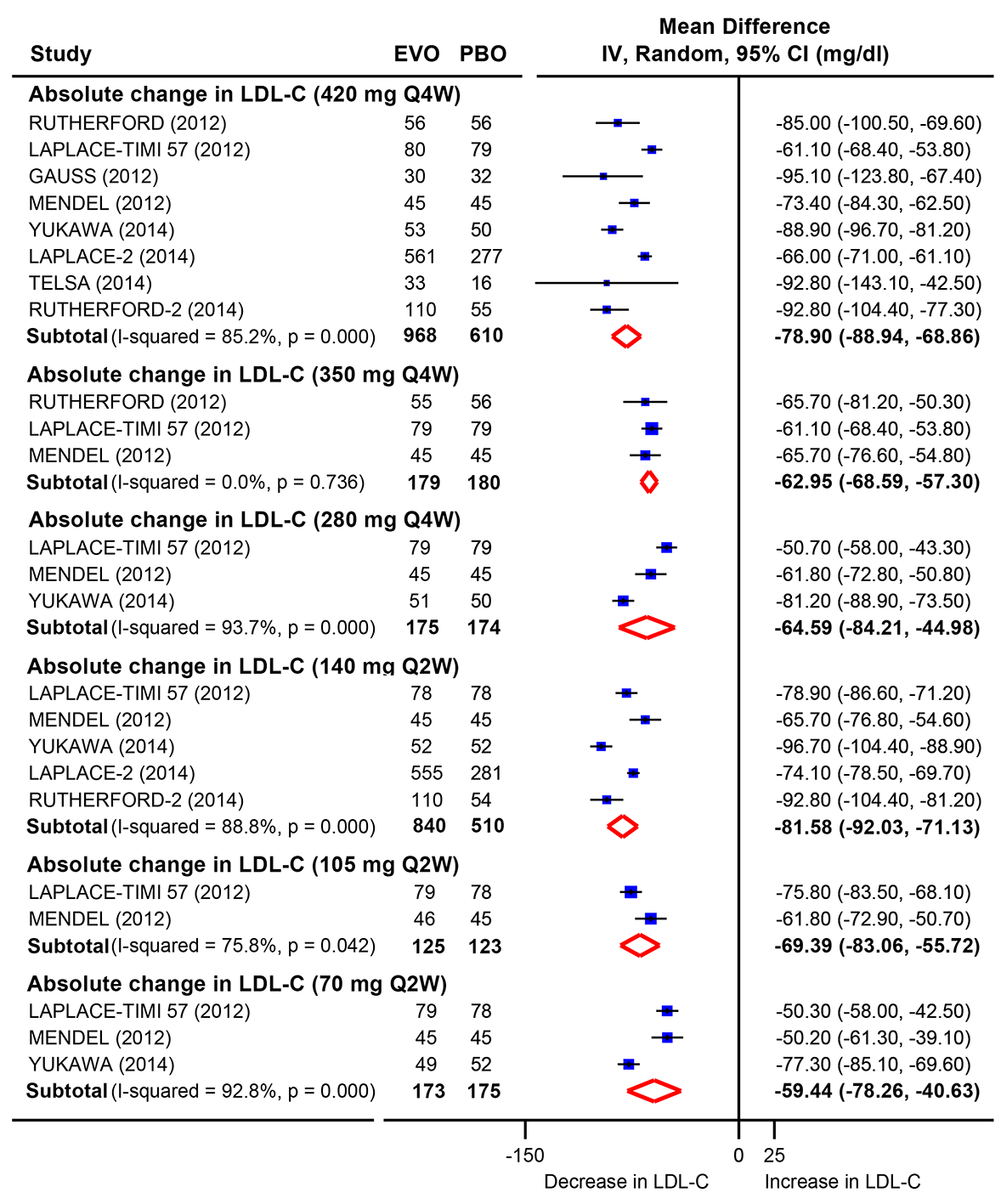


**Figure S1. Forest plot demonstrating absolute changes in LDL cholesterol (LDL-C) stratified by dosages following evolocumab treatments versus placebo at 12 weeks follow-up.** CI = confidence interval; EVO = evolocumab; PBO = placebo; TC = total cholesterol. Other abbreviations as in Table S1.

**Table S4. Percent and absolute changes of LDL cholesterol (LDL-C) at week 12 after evolocumab treatment versus placebo or ezetimibe.**

| **EVO dose** | **Mean Difference (95% CI)** | **Test for overall effect** | | **No. of studies** | **No. of individuals** | | **Heterogeneity** | | **Publication bias** | |
| --- | --- | --- | --- | --- | --- | --- | --- | --- | --- | --- |
|  |  | ***Z*** | ***P* value** |  | **PBO/EZE** | **EVO** | ***I^2^*** | ***P* value** | ***P*_Begg** | ***P*_Egger** |
| % change in LDL-C at 12 weeks follow-up (EVO vs. PBO) | | | | | | | | | | |
| 420 mg Q4W | **-54.61 (-58.67, -50.54)** | 26.32 | 0.000 | 10 | 990 | 1720 | 80.4% | 0.000 | 0.371 | 0.138 |
| 350 mg Q4W | **-47.75 (-51.59, -43.90)** | 24.34 | 0.000 | 3 | 180 | 179 | 0.0% | 0.441 | 0.296 | 0.245 |
| 280 mg Q4W | **-47.87 (-58.28, -37.47)** | 9.02 | 0.000 | 3 | 174 | 175 | 87.5% | 0.000 | 1.000 | 0.932 |
| 140 mg Q2W | **-60.39 (-68.77, -52.02)** | 14.13 | 0.000 | 6 | 586 | 993 | 93.9% | 0.000 | 0.707 | 0.480 |
| 105 mg Q2W | **-50.35 (-69.95, -30.75)** | 5.04 | 0.000 | 2 | 123 | 125 | 94.8% | 0.000 | 1.000 | NA |
| 70 mg Q2W | **-44.14 (-52.99, -35.29)** | 9.77 | 0.000 | 3 | 175 | 173 | 84.1% | 0.000 | 1.000 | 0.789 |
| Absolute change in LDL-C at 12 weeks follow-up (EVO vs. PBO, mg/dl) | | | | | | | | | | |
| 420 mg Q4W | **-78.90 (-88.94, -68.86)** | 15.40 | 0.000 | 8 | 610 | 968 | 85.2% | 0.000 | 0.536 | 0.144 |
| 350 mg Q4W | **-62.95 (-68.59, -57.30)** | 21.85 | 0.000 | 3 | 180 | 179 | 0.0% | 0.736 | 1.000 | 0.314 |
| 280 mg Q4W | **-64.59 (-84.21, -44.98)** | 6.45 | 0.000 | 3 | 174 | 175 | 93.7% | 0.000 | 1.000 | 0.997 |
| 140 mg Q2W | **-81.58 (-92.03, -71.13)** | 15.30 | 0.000 | 5 | 510 | 840 | 88.8% | 0.000 | 0.462 | 0.524 |
| 105 mg Q2W | **-69.39 (-83.06, -55.72)** | 9.95 | 0.000 | 2 | 123 | 125 | 75.8% | 0.042 | 1.000 | NA |
| 70 mg Q2W | **-59.44 (-78.26, -40.63)** | 6.19 | 0.000 | 3 | 175 | 173 | 92.8% | 0.000 | 1.000 | 0.709 |
| % change in LDL-C at 12 weeks follow-up (EVO vs. EZE) | | | | | | | | | | |
| 420 mg Q4W | **-36.30 (-38.75, -33.85)** | 29.01 | 0.000 | 5 | 314 | 552 | 0.0% | 0.494 | 0.806 | 0.912 |
| 350 mg Q4W | **-28.73 (-33.74, -23.72)** | 11.24 | 0.000 | 2 | 77 | 76 | 0.0% | 0.776 | 1.000 | NA |
| 280 mg Q4W | **-25.50 (-30.47, -20.53)** | 10.05 | 0.000 | 2 | 77 | 77 | 0.0% | 0.879 | 1.000 | NA |
| 140 mg Q2W | **-38.19 (-41.51, -34.88)** | 22.61 | 0.000 | 4 | 285 | 520 | 28.4% | 0.242 | 0.734 | 0.504 |
| 105 mg Q2W | **-29.60 (-36.70, -22.50)** | 8.17 | 0.000 | 1 | 45 | 46 | NA | NA | NA | NA |
| 70 mg Q2W | **-26.70 (-33.85, -19.55)** | 7.32 | 0.000 | 1 | 45 | 45 | NA | NA | NA | NA |
| Absolute change in LDL-C at 12 weeks follow-up (EVO vs. EZE, mg/dl) | | | | | | | | | | |
| 420 mg Q4W | **-61.22 (-82.47, -39.97)** | 5.65 | 0.000 | 3 | 192 | 354 | 89.3% | 0.000 | 1.000 | 0.488 |
| 350 mg Q4W | **-55.50 (-80.40, -30.60)** | 4.37 | 0.000 | 1 | 32 | 31 | NA | NA | NA | NA |
| 280 mg Q4W | **-52.60 (-77.30, -27.90)** | 4.17 | 0.000 | 1 | 32 | 32 | NA | NA | NA | NA |
| 140 mg Q2W | **-58.28 (-79.62, -36.94)** | 5.35 | 0.000 | 2 | 163 | 322 | 88.4% | 0.003 | 1.000 | NA |

CI = confidence interval; EVO = evolocumab; EZE = ezetimibe; LDL-C = low-density lipoprotein (LDL) cholesterol; NA = not applicable; PBO = placebo.

**Table S5. Percent and absolute changes of LDL cholesterol at mean of weeks 10 and 12 after evolocumab treatment versus placebo or ezetimibe**.

| **EVO dose** | **Mean Difference (95% CI)** | **Test for overall effect** | | **No. of studies** | **No. of individuals** | | **Heterogeneity** | | **Publication bias** | |
| --- | --- | --- | --- | --- | --- | --- | --- | --- | --- | --- |
|  |  | ***Z*** | ***P* value** |  | **PBO/EZE** | **EVO** | ***I^2^*** | ***P* value** | ***P*_Begg** | ***P*_Egger** |
| % change in LDL-C at mean of weeks 10 and 12 (EVO vs. PBO) | | | | | | | | | | |
| 420 mg Q4W | **-59.17 (-67.16, -51.18)** | 14.51 | 0.000 | 5 | 476 | 910 | 92.9% | 0.000 | 0.312 | 0.434 |
| 140 mg Q2W | **-64.61 (-71.97, -57.24)** | 17.20 | 0.000 | 4 | 463 | 870 | 94.5% | 0.000 | 0.308 | 0.356 |
| Absolute change in LDL-C at mean of weeks 10 and 12 (EVO vs. PBO, mg/dl) | | | | | | | | | | |
| 420 mg Q4W | **-71.13 (-75.75, -66.51)** | 30.18 | 0.000 | 2 | 293 | 594 | 0.0% | 0.388 | 1.000 | NA |
| 140 mg Q2W | **-72.60 (-76.75, -68.45)** | 34.29 | 0.000 | 1 | 281 | 555 | NA | NA | NA | NA |
| % change in LDL-C at mean of weeks 10 and 12 (EVO vs. EZE) | | | | | | | | | | |
| 420 mg Q4W | **-40.30 (-42.86, -37.74)** | 30.87 | 0.000 | 3 | 237 | 475 | 13.3% | 0.315 | 1.000 | 0.571 |
| 140 mg Q2W | **-39.23 (-41.80, -36.66)** | 29.86 | 0.000 | 3 | 240 | 475 | 0.0% | 0.536 | 1.000 | 0.942 |
| Absolute change in LDL-C at mean of weeks 10 and 12 (EVO vs. EZE, mg/dl) | | | | | | | | | | |
| 420 mg Q4W | **-58.47 (-81.69, -35.25)** | 4.94 | 0.000 | 2 | 160 | 322 | 93.3% | 0.000 | 1.000 | NA |
| 140 mg Q2W | **-55.21 (-75.96, -34.46)** | 5.21 | 0.000 | 2 | 163 | 322 | 89.0% | 0.003 | 1.000 | NA |

CI = confidence interval; EVO = evolocumab; EZE = ezetimibe; LDL-C = low-density lipoprotein (LDL) cholesterol; NA = not applicable; PBO = placebo.

**Table S6. Percent change of HDL cholesterol at week 12 and at mean of weeks 10 and 12 after evolocumab treatment versus placebo or ezetimibe**.

| **EVO dose** | **Mean Difference (95% CI)** | **Test for overall effect** | | **No. of studies** | **No. of individuals** | | **Heterogeneity** | | **Publication bias** | |
| --- | --- | --- | --- | --- | --- | --- | --- | --- | --- | --- |
|  |  | ***Z*** | ***P* value** |  | **PBO/EZE** | **EVO** | ***I^2^*** | ***P* value** | ***P*_Begg** | ***P*_Egger** |
| % change in HDL-C at 12 weeks follow-up (EVO vs. PBO) | | | | | | | | | | |
| 420 mg Q4W | **7.58 (5.69, 9.46)** | 7.89 | 0.000 | 9 | 688 | 1121 | 23.3% | 0.236 | 1.000 | 0.843 |
| 350 mg Q4W | **5.97 (3.08, 8.86)** | 4.04 | 0.000 | 3 | 180 | 179 | 0.0% | 0.648 | 1.000 | 0.824 |
| 280 mg Q4W | 6.96 (-2.19, 16.11) | 1.49 | 0.136 | 3 | 174 | 175 | 87.4% | 0.000 | 1.000 | 0.641 |
| 140 mg Q2W | **6.90 (5.37, 8.43)** | 8.84 | 0.000 | 6 | 586 | 993 | 0.0% | 0.551 | 0.133 | 0.030 |
| 105 mg Q2W | **6.83 (3.08, 10.59)** | 3.57 | 0.000 | 2 | 123 | 125 | 0.0% | 0.863 | 1.000 | NA |
| 70 mg Q2W | **5.58 (2.32, 8.83)** | 3.36 | 0.001 | 3 | 175 | 173 | 0.0% | 0.736 | 0.296 | 0.050 |
| % change in HDL-C at mean of weeks 10 and 12 (EVO vs. PBO) | | | | | | | | | | |
| 420 mg Q4W | **8.59 (6.22, 10.96)** | 7.10 | 0.000 | 5 | 476 | 910 | 50.9% | 0.087 | 0.806 | 0.919 |
| 280 mg Q4W | **16.40 (11.30, 21.50)** | 6.30 | 0.000 | 1 | 50 | 51 | NA | NA | NA | NA |
| 140 mg Q2W | **6.64 (5.20, 8.08)** | 9.04 | 0.000 | 4 | 463 | 870 | 0.0% | 0.513 | 0.308 | 0.302 |
| 70 mg Q2W | **6.40 (1.10, 11.70)** | 2.37 | 0.018 | 1 | 52 | 49 | NA | NA | NA | NA |
| % change in HDL-C at 12 weeks follow-up (EVO vs. EZE) | | | | | | | | | | |
| 420 mg Q4W | **6.35 (4.26, 8.44)** | 5.95 | 0.000 | 4 | 269 | 507 | 0.0% | 0.701 | 1.000 | 0.874 |
| 350 mg Q4W | 6.60 (-3.10, 16.30) | 1.33 | 0.182 | 1 | 32 | 31 | NA | NA | NA | NA |
| 280 mg Q4W | 7.00 (-2.70, 16.70) | 1.41 | 0.157 | 1 | 32 | 32 | NA | NA | NA | NA |
| 140 mg Q2W | **7.19 (4.39, 9.99)** | 5.04 | 0.000 | 3 | 240 | 475 | 32.2% | 0.229 | 0.296 | 0.334 |
| % change in HDL-C at mean of weeks 10 and 12 (EVO vs. EZE) | | | | | | | | | | |
| 420 mg Q4W | **6.18 (3.27, 9.10)** | 4.16 | 0.000 | 3 | 237 | 475 | 43.6% | 0.170 | 0.296 | 0.187 |
| 140 mg Q2W | **6.17 (4.15, 8.18)** | 6.01 | 0.000 | 3 | 240 | 475 | 0.0% | 0.553 | 1.000 | 0.190 |

CI = confidence interval; EVO = evolocumab; EZE = ezetimibe; HDL-C = high-density lipoprotein (HDL) cholesterol; NA = not applicable; PBO = placebo.


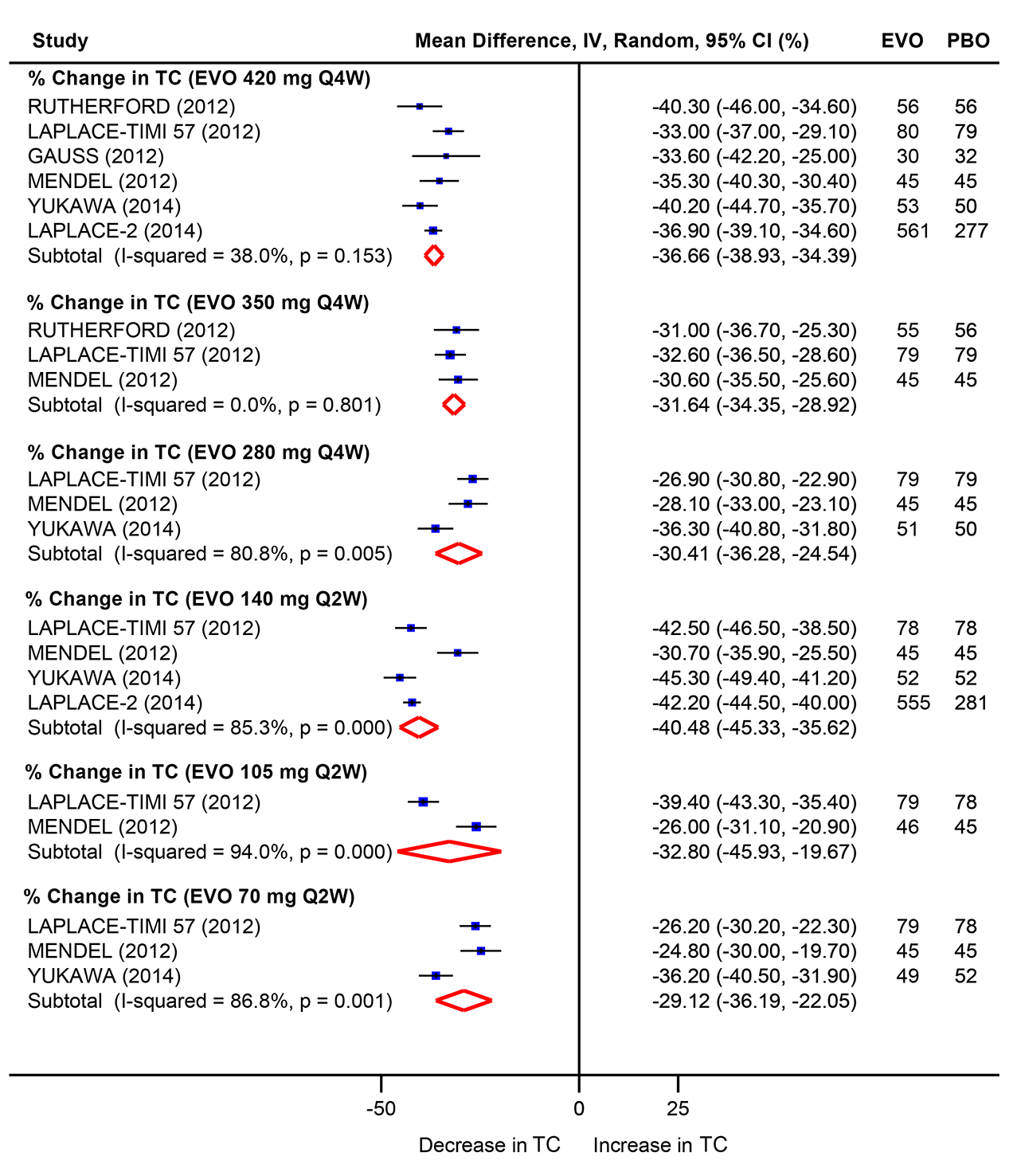


**Figure S2. Forest plot demonstrating changes in total cholesterol (TC) stratified by dosages following evolocumab treatments versus placebo at 12 weeks follow-up.** CI = confidence interval; EVO = evolocumab; PBO = placebo; TC = total cholesterol. Other abbreviations as in Table S1.

.

**Table S7. Percent change of total cholesterol at week 12 and at mean of weeks 10 and 12 after evolocumab treatment versus placebo or ezetimibe**.

| **EVO dose** | **Mean Difference (95% CI)** | **Test for overall effect** | | **No. of studies** | **No. of individuals** | | **Heterogeneity** | | **Publication bias** | |
| --- | --- | --- | --- | --- | --- | --- | --- | --- | --- | --- |
|  |  | ***Z*** | ***P* value** |  | **PBO/EZE** | **EVO** | ***I^2^*** | ***P* value** | ***P*_Begg** | ***P*_Egger** |
| % change in TC at 12 weeks follow-up (EVO vs. PBO) | | | | | | | | | | |
| 420 mg Q4W | **-36.66 (-38.93, -34.39)** | 31.60 | 0.000 | 6 | 539 | 825 | 38.0% | 0.153 | 1.000 | 0.980 |
| 350 mg Q4W | **-31.64 (-34.35, -28.92)** | 22.84 | 0.000 | 3 | 180 | 179 | 0.0% | 0.801 | 1.000 | 0.337 |
| 280 mg Q4W | **-30.41 (-36.28, -24.54)** | 10.15 | 0.000 | 3 | 174 | 175 | 80.8% | 0.005 | 1.000 | 0.839 |
| 140 mg Q2W | **-40.48 (-45.33, -35.62)** | 16.35 | 0.000 | 4 | 456 | 730 | 85.3% | 0.000 | 0.734 | 0.552 |
| 105 mg Q2W | **-32.80 (-45.93, -19.67)** | 4.90 | 0.000 | 2 | 123 | 125 | 94.0% | 0.000 | 1.000 | NA |
| 70 mg Q2W | **-29.12 (-36.19, -22.05)** | 8.08 | 0.000 | 3 | 175 | 173 | 86.8% | 0.001 | 1.000 | 0.898 |
| % change in TC at mean of weeks 10 and 12 (EVO vs. PBO) | | | | | | | | | | |
| 420 mg Q4W | **-40.29 (-42.21, -38.36)** | 41.07 | 0.000 | 2 | 327 | 614 | 0.0% | 0.415 | 1.000 | NA |
| 280 mg Q4W | **-39.80 (-43.50, -36.10)** | 21.08 | 0.000 | 1 | 50 | 51 | NA | NA | NA | NA |
| 140 mg Q2W | **-43.21 (-47.39, -39.02)** | 20.23 | 0.000 | 2 | 333 | 607 | 76.8% | 0.038 | 1.000 | NA |
| 70 mg Q2W | **-36.20 (-39.70, -32.70)** | 20.27 | 0.000 | 1 | 52 | 49 | NA | NA | NA | NA |
| % change in TC at 12 weeks follow-up (EVO vs. EZE) | | | | | | | | | | |
| 420 mg Q4W | **-23.53 (-27.08, -19.99)** | 13.01 | 0.000 | 2 | 141 | 252 | 0.0% | 0.379 | 1.000 | NA |
| 350 mg Q4W | **-19.70 (-28.20, -11.20)** | 4.54 | 0.000 | 1 | 32 | 31 | NA | NA | NA | NA |
| 280 mg Q4W | **-19.10 (-27.60, -10.60)** | 4.40 | 0.000 | 1 | 32 | 32 | NA | NA | NA | NA |
| 140 mg Q2W | **-24.50 (-28.55, -20.45)** | 11.86 | 0.000 | 1 | 112 | 219 | NA | NA | NA | NA |
| % change in TC at mean of weeks 10 and 12 (EVO vs. EZE) | | | | | | | | | | |
| 420 mg Q4W | **-25.00 (-28.50, -21.50)** | 14.00 | 0.000 | 1 | 109 | 220 | NA | NA | NA | NA |
| 140 mg Q2W | **-23.30 (-27.00, -19.60)** | 12.34 | 0.000 | 1 | 112 | 219 | NA | NA | NA | NA |

CI = confidence interval; EVO = evolocumab; EZE = ezetimibe; NA = not applicable; PBO = placebo; TC = total cholesterol.


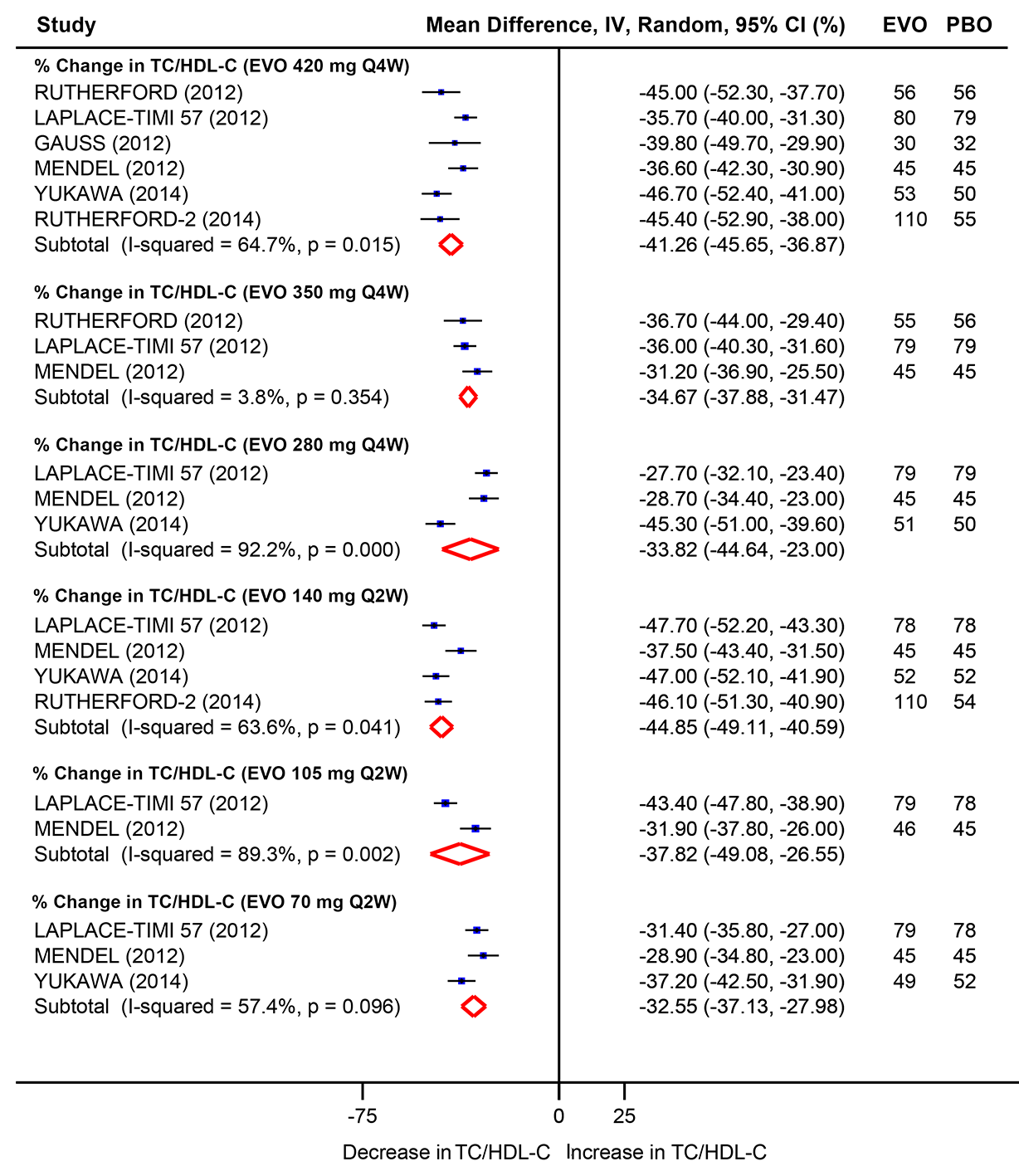


**Figure S3. Forest plot demonstrating changes in total cholesterol/HDL-C ratio stratified by dosages following evolocumab treatments versus placebo at 12 weeks follow-up.** CI = confidence interval; EVO = evolocumab; PBO = placebo; TC/HDL-C = total cholesterol/high-density lipoprotein cholesterol ratio. Other abbreviations as in Table S1.

**Table S8. Percent change of total cholesterol/HDL-C ratio at week 12 and at mean of weeks 10 and 12 after evolocumab treatment versus placebo or ezetimibe**.

| **EVO dose** | **Mean Difference (95% CI)** | **Test for overall effect** | | **No. of studies** | **No. of individuals** | | **Heterogeneity** | | **Publication bias** | |
| --- | --- | --- | --- | --- | --- | --- | --- | --- | --- | --- |
|  |  | ***Z*** | ***P* value** |  | **PBO/EZE** | **EVO** | ***I^2^*** | ***P* value** | ***P*_Begg** | ***P*_Egger** |
| % change in TC/HDL-C at 12 weeks follow-up (EVO vs. PBO) | | | | | | | | | | |
| 420 mg Q4W | **-41.26 (-45.65, -36.87)** | 18.43 | 0.000 | 6 | 317 | 374 | 64.7% | 0.015 | 0.566 | 0.302 |
| 350 mg Q4W | **-34.67 (-37.88, -31.47)** | 21.21 | 0.000 | 3 | 180 | 179 | 3.8% | 0.354 | 1.000 | 0.926 |
| 280 mg Q4W | **-33.82 (-44.64, -23.00)** | 6.13 | 0.000 | 3 | 174 | 175 | 92.2% | 0.000 | 0.540 | 0.583 |
| 140 mg Q2W | **-44.85 (-49.11, -40.59)** | 20.64 | 0.000 | 4 | 229 | 285 | 63.6% | 0.041 | 0.089 | 0.126 |
| 105 mg Q2W | **-37.82 (-49.08, -26.55)** | 6.58 | 0.000 | 2 | 123 | 125 | 89.3% | 0.002 | 1.000 | NA |
| 70 mg Q2W | **-32.55 (-37.13, -27.98)** | 13.96 | 0.000 | 3 | 175 | 173 | 57.4% | 0.096 | 1.000 | 0.974 |
| % change in TC/HDL-C at mean of weeks 10 and 12 (EVO vs. PBO) | | | | | | | | | | |
| 420 mg Q4W | **-49.20 (-54.25, -44.15)** | 19.10 | 0.000 | 1 | 55 | 110 | NA | NA | NA | NA |
| 140 mg Q2W | **-46.60 (-51.40, -41.80)** | 19.03 | 0.000 | 1 | 54 | 110 | NA | NA | NA | NA |
| % change in TC/HDL-C at 12 weeks follow-up (EVO vs. EZE) | | | | | | | | | | |
| 420 mg Q4W | **-29.24 (-33.83, -24.64)** | 12.47 | 0.000 | 2 | 83 | 134 | 0.0% | 0.678 | 1.000 | NA |
| 350 mg Q4W | **-23.90 (-32.90, -14.90)** | 5.20 | 0.000 | 1 | 32 | 31 | NA | NA | NA | NA |
| 280 mg Q4W | **-22.40 (-31.30, -13.50)** | 4.93 | 0.000 | 1 | 32 | 32 | NA | NA | NA | NA |
| 140 mg Q2W | **-26.30 (-31.40, -21.20)** | 10.11 | 0.000 | 1 | 51 | 103 | NA | NA | NA | NA |
| % change in TC/HDL-C at mean of weeks 10 and 12 (EVO vs. EZE) | | | | | | | | | | |
| 420 mg Q4W | **-29.90 (-34.65, -25.15)** | 12.34 | 0.000 | 1 | 51 | 102 | NA | NA | NA | NA |
| 140 mg Q2W | **-27.40 (-32.10, -22.70)** | 11.43 | 0.000 | 1 | 51 | 103 | NA | NA | NA | NA |

CI = confidence interval; EVO = evolocumab; EZE = ezetimibe; NA = not applicable; PBO = placebo; TC/HDL-C = total cholesterol/high-density lipoprotein cholesterol ratio.


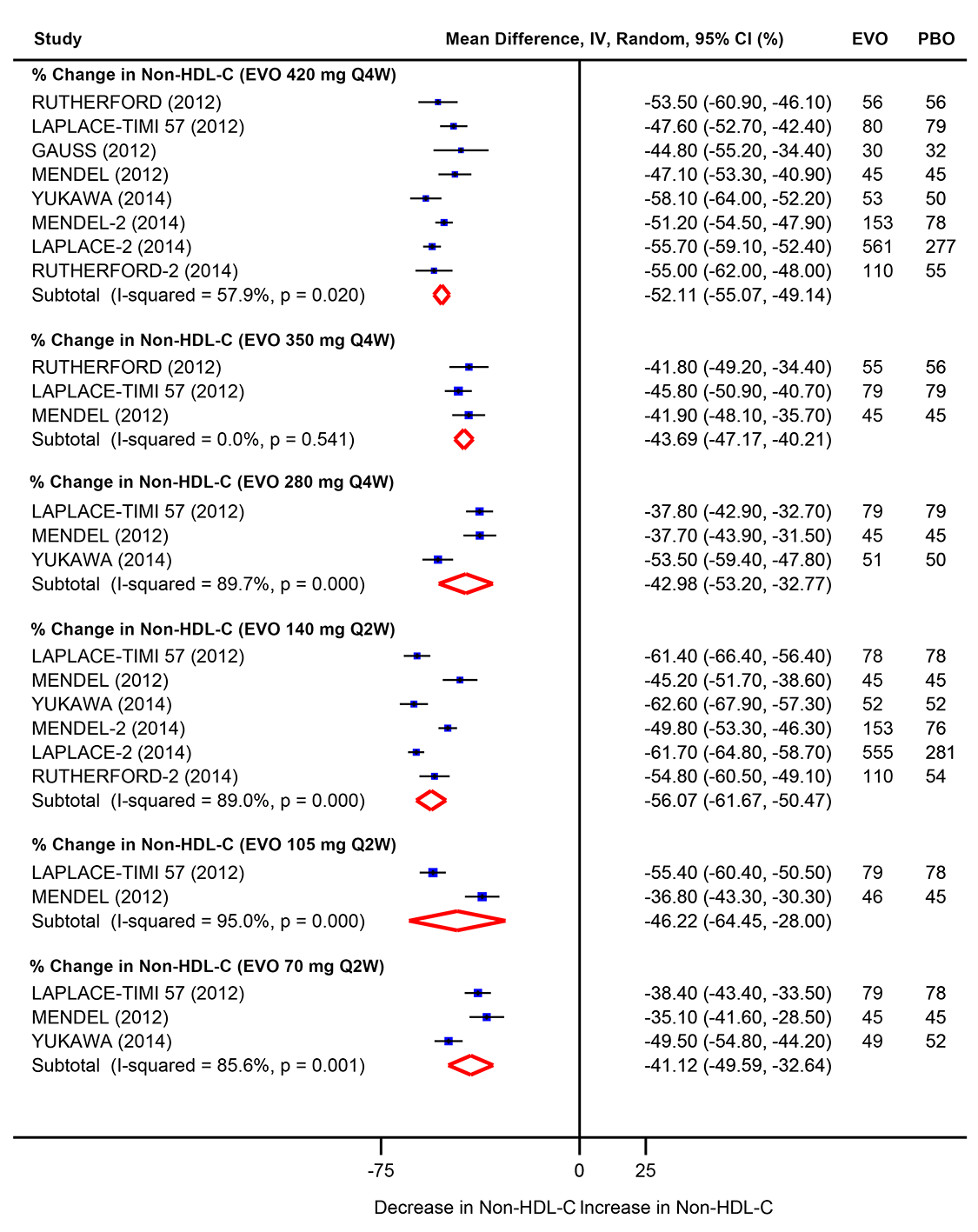


**Figure S4. Forest plot demonstrating changes in Non-HDL cholesterol (Non-HDL-C) stratified by dosages following evolocumab treatments versus placebo at 12 weeks follow-up.** CI = confidence interval; EVO = evolocumab; Non-HDL-C = Non-high-density lipoprotein (Non-HDL) cholesterol; PBO = placebo. Other abbreviations as in Table S1.

**Table S9. Percent change of Non-HDL cholesterol at week 12 and at mean of weeks 10 and 12 after evolocumab treatment versus placebo or ezetimibe**.

| **EVO dose** | **Mean Difference (95% CI)** | **Test for overall effect** | | **No. of studies** | **No. of individuals** | | **Heterogeneity** | | **Publication bias** | |
| --- | --- | --- | --- | --- | --- | --- | --- | --- | --- | --- |
|  |  | ***Z*** | ***P* value** |  | **PBO/EZE** | **EVO** | ***I^2^*** | ***P* value** | ***P*_Begg** | ***P*_Egger** |
| % change in Non-HDL-C at 12 weeks follow-up (EVO vs. PBO) | | | | | | | | | | |
| 420 mg Q4W | **-52.11 (-55.07, -49.14)** | 34.42 | 0.000 | 8 | 672 | 1088 | 57.9% | 0.020 | 0.618 | 0.499 |
| 350 mg Q4W | **-43.69 (-47.17, -40.21)** | 24.63 | 0.000 | 3 | 180 | 179 | 0.0% | 0.541 | 0.296 | 0.264 |
| 280 mg Q4W | **-42.98 (-53.20, -32.77)** | 8.25 | 0.000 | 3 | 174 | 175 | 89.7% | 0.000 | 1.000 | 0.894 |
| 140 mg Q2W | **-56.07 (-61.67, -50.47)** | 19.62 | 0.000 | 6 | 586 | 993 | 89.0% | 0.000 | 0.452 | 0.616 |
| 105 mg Q2W | **-46.22 (-64.45, -28.00)** | 4.97 | 0.000 | 2 | 123 | 125 | 95.0% | 0.000 | 1.000 | NA |
| 70 mg Q2W | **-41.12 (-49.59, -32.64)** | 9.51 | 0.000 | 3 | 175 | 173 | 85.6% | 0.001 | 1.000 | 0.766 |
| % change in Non-HDL-C at mean of weeks 10 and 12 (EVO vs. PBO) | | | | | | | | | | |
| 420 mg Q4W | **-58.23 (-62.18, -54.28)** | 28.91 | 0.000 | 4 | 460 | 877 | 77.4% | 0.004 | 1.000 | 0.571 |
| 280 mg Q4W | **-58.30 (-62.80, -53.80)** | 25.39 | 0.000 | 1 | 50 | 51 | NA | NA | NA | NA |
| 140 mg Q2W | **-57.10 (-63.86, -50.34)** | 16.35 | 0.000 | 4 | 463 | 870 | 92.5% | 0.000 | 0.734 | 0.930 |
| 70 mg Q2W | **-50.10 (-54.40, -45.80)** | 22.84 | 0.000 | 1 | 52 | 49 | NA | NA | NA | NA |
| % change in Non-HDL-C at 12 weeks follow-up (EVO vs. EZE) | | | | | | | | | | |
| 420 mg Q4W | **-33.54 (-35.81, -31.27)** | 28.91 | 0.000 | 4 | 269 | 507 | 0.0% | 0.914 | 0.734 | 0.576 |
| 350 mg Q4W | **-26.60 (-36.60, -16.60)** | 5.21 | 0.000 | 1 | 32 | 31 | NA | NA | NA | NA |
| 280 mg Q4W | **-24.80 (-34.80, -14.80)** | 4.86 | 0.000 | 1 | 32 | 32 | NA | NA | NA | NA |
| 140 mg Q2W | **-35.03 (-37.77, -32.28)** | 25.01 | 0.000 | 3 | 240 | 475 | 10.7% | 0.326 | 1.000 | 0.993 |
| % change in Non-HDL-C at mean of weeks 10 and 12 (EVO vs. EZE) | | | | | | | | | | |
| 420 mg Q4W | **-35.84 (-37.98, -33.71)** | 32.90 | 0.000 | 3 | 237 | 475 | 0.0% | 0.451 | 1.000 | 0.615 |
| 140 mg Q2W | **-34.58 (-37.00, -32.16)** | 28.02 | 0.000 | 3 | 240 | 475 | 5.1% | 0.349 | 1.000 | 0.603 |

CI = confidence interval; EVO = evolocumab; EZE = ezetimibe; Non-HDL-C = Non-high-density lipoprotein (Non-HDL) cholesterol; NA = not applicable; PBO = placebo.


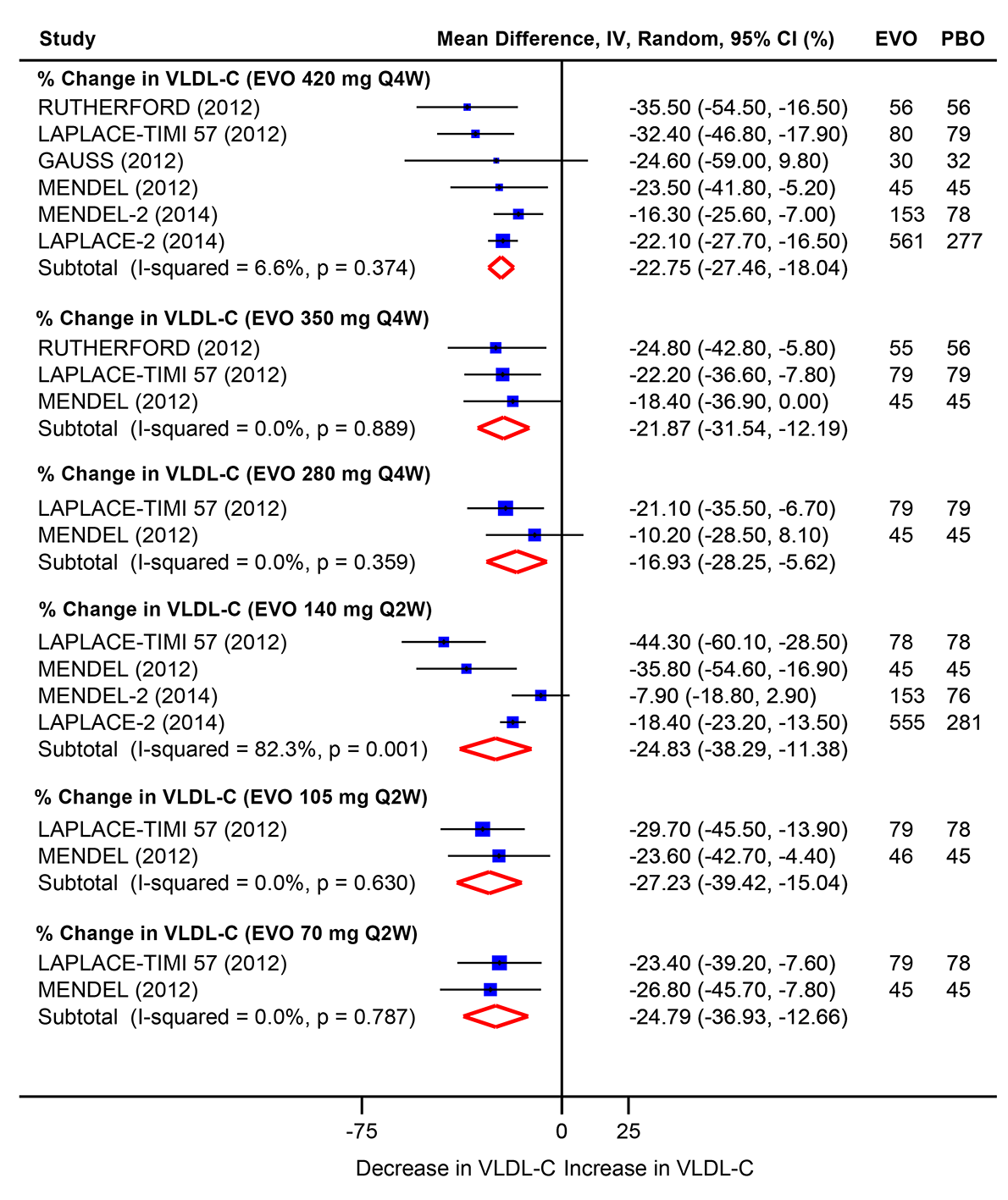


**Figure S5. Forest plot demonstrating changes in VLDL cholesterol (VLDL-C) stratified by dosages following evolocumab treatments versus placebo at 12 weeks follow-up.** CI = confidence interval; EVO = evolocumab; PBO = placebo; VLDL-C = very low-density lipoprotein (VLDL) cholesterol. Other abbreviations as in Table S1.

**Table S10. Percent change of VLDL cholesterol (VLDL-C) at week 12 and at mean of weeks 10 and 12 after evolocumab treatment versus placebo or ezetimibe.**

| **EVO dose** | **Mean Difference (95% CI)** | **Test for overall effect** | | **No. of studies** | **No. of individuals** | | **Heterogeneity** | | **Publication bias** | |
| --- | --- | --- | --- | --- | --- | --- | --- | --- | --- | --- |
|  |  | ***Z*** | ***P* value** |  | **PBO/EZE** | **EVO** | ***I^2^*** | ***P* value** | ***P*_Begg** | ***P*_Egger** |
| % change in VLDL-C at 12 weeks follow-up (EVO vs. PBO) | | | | | | | | | | |
| 420 mg Q4W | **-22.75 (-27.46, -18.04)** | 9.47 | 0.000 | 6 | 567 | 925 | 6.6% | 0.374 | 0.452 | 0.335 |
| 350 mg Q4W | **-21.87 (-31.54, -12.19)** | 4.43 | 0.000 | 3 | 180 | 179 | 0.0% | 0.889 | 1.000 | 0.971 |
| 280 mg Q4W | **-16.93 (-28.25, -5.62)** | 2.93 | 0.003 | 2 | 124 | 124 | 0.0% | 0.359 | 1.000 | NA |
| 140 mg Q2W | **-24.83 (-38.29, -11.38)** | 3.62 | 0.000 | 4 | 480 | 831 | 82.3% | 0.001 | 0.734 | 0.462 |
| 105 mg Q2W | **-27.23 (-39.42, -15.04)** | 4.38 | 0.000 | 2 | 123 | 125 | 0.0% | 0.630 | 1.000 | NA |
| 70 mg Q2W | **-24.79 (-36.93, -12.66)** | 4.00 | 0.000 | 2 | 123 | 124 | 0.0% | 0.787 | 1.000 | NA |
| % change in VLDL-C at mean of weeks 10 and 12 (EVO vs. PBO) | | | | | | | | | | |
| 420 mg Q4W | **-22.11 (-26.30, -17.91)** | 10.33 | 0.000 | 2 | 355 | 714 | 0.0% | 0.702 | 1.000 | NA |
| 140 mg Q2W | -11.90 (-25.71, 1.91) | 1.69 | 0.091 | 2 | 357 | 708 | 91.8% | 0.000 | 1.000 | NA |
| % change in VLDL-C at 12 weeks follow-up (EVO vs. EZE) | | | | | | | | | | |
| 420 mg Q4W | **-6.76 (-12.86, -0.65)** | 2.17 | 0.030 | 4 | 269 | 507 | 17.3% | 0.305 | 0.734 | 0.519 |
| 350 mg Q4W | -15.60 (-45.50, 14.30) | 1.02 | 0.307 | 1 | 32 | 31 | NA | NA | NA | NA |
| 280 mg Q4W | -14.40 (-44.10, 15.30) | 0.95 | 0.342 | 1 | 32 | 32 | NA | NA | NA | NA |
| 140 mg Q2W | -3.76 (-9.36, 1.84) | 1.32 | 0.188 | 3 | 240 | 475 | 0.0% | 0.461 | 1.000 | 0.895 |
| % change in VLDL-C at mean of weeks 10 and 12 (EVO vs. EZE) | | | | | | | | | | |
| 420 mg Q4W | **-9.91 (-15.40, -4.42)** | 3.54 | 0.000 | 3 | 237 | 475 | 17.3% | 0.298 | 0.296 | 0.608 |
| 140 mg Q2W | -2.47 (-6.87, 1.92) | 1.10 | 0.270 | 3 | 240 | 475 | 0.0% | 0.669 | 1.000 | 0.546 |

CI = confidence interval; EVO = evolocumab; EZE = ezetimibe; NA = not applicable; PBO = placebo; VLDL-C = very low-density lipoprotein (VLDL) cholesterol.


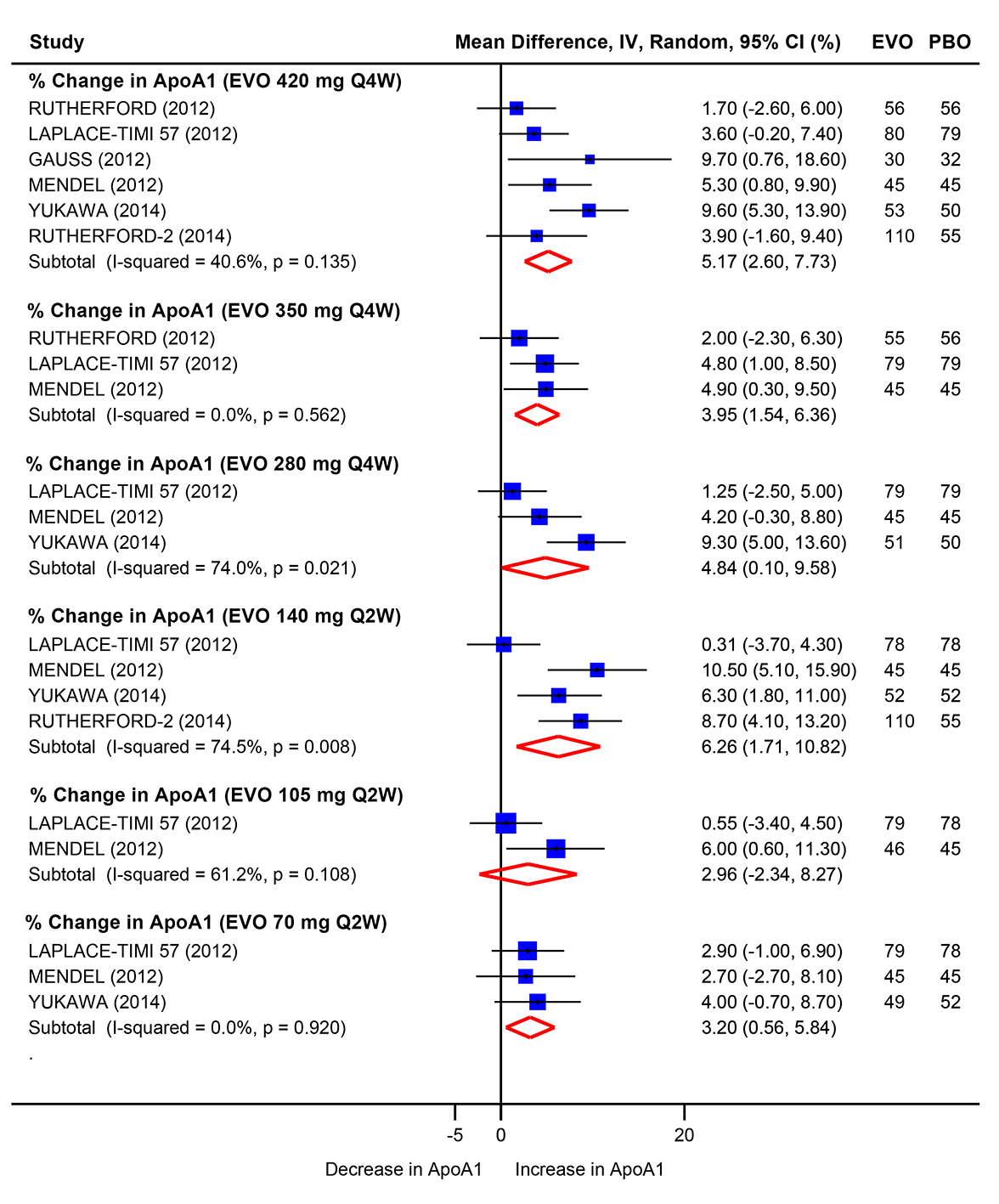


**Figure S6. Forest plot demonstrating changes in apolipoprotein A1 (ApoA1) stratified by dosages following evolocumab treatments versus placebo at 12 weeks follow-up.** CI = confidence interval; ApoA1 = apolipoprotein A1; EVO = evolocumab; PBO = placebo. Other abbreviations as in Table S1.

**Table S11. Percent change of apolipoprotein A1 (ApoA1) at week 12 and at mean of weeks 10 and 12 after evolocumab treatment versus placebo or ezetimibe**.

| **EVO dose** | **Mean Difference (95% CI)** | **Test for overall effect** | | **No. of studies** | **No. of individuals** | | **Heterogeneity** | | **Publication bias** | |
| --- | --- | --- | --- | --- | --- | --- | --- | --- | --- | --- |
|  |  | ***Z*** | ***P* value** |  | **PBO/EZE** | **EVO** | ***I^2^*** | ***P* value** | ***P*_Begg** | ***P*_Egger** |
| % change in ApoA1 at 12 weeks follow-up (EVO vs. PBO) | | | | | | | | | | |
| 420 mg Q4W | **5.17 (2.60, 7.73)** | 3.95 | 0.000 | 6 | 317 | 374 | 40.6% | 0.135 | 0.566 | 0.517 |
| 350 mg Q4W | **3.95 (1.54, 6.36)** | 3.21 | 0.001 | 3 | 180 | 179 | 0.0% | 0.562 | 1.000 | 0.836 |
| 280 mg Q4W | **4.84 (0.10, 9.58)** | 2.00 | 0.045 | 3 | 174 | 175 | 74.0% | 0.021 | 1.000 | 0.602 |
| 140 mg Q2W | **6.26 (1.71, 10.82)** | 2.69 | 0.007 | 4 | 230 | 285 | 74.5% | 0.008 | 0.308 | 0.129 |
| 105 mg Q2W | 2.96 (-2.34, 8.27) | 1.09 | 0.274 | 2 | 123 | 125 | 61.2% | 0.108 | 1.000 | NA |
| 70 mg Q2W | **3.20 (0.56, 5.84)** | 2.38 | 0.017 | 3 | 175 | 173 | 0.0% | 0.920 | 1.000 | 0.982 |
| % change in ApoA1 at mean of weeks 10 and 12 (EVO vs. PBO) | | | | | | | | | | |
| 420 mg Q4W | **7.34 (2.84, 11.84)** | 3.20 | 0.001 | 2 | 105 | 163 | 60.4% | 0.112 | 1.000 | NA |
| 280 mg Q4W | **10.60 (6.90, 14.30)** | 5.62 | 0.000 | 1 | 50 | 51 | NA | NA | NA | NA |
| 140 mg Q2W | **6.74 (3.87, 9.61)** | 4.60 | 0.000 | 2 | 106 | 162 | 0.0% | 0.713 | 1.000 | NA |
| 70 mg Q2W | **5.00 (1.30, 8.70)** | 2.65 | 0.008 | 1 | 52 | 49 | NA | NA | NA | NA |
| % change in ApoA1 at 12 weeks follow-up (EVO vs. EZE) | | | | | | | | | | |
| 420 mg Q4W | 4.79 (-1.48, 11.06) | 1.50 | 0.134 | 2 | 83 | 134 | 51.8% | 0.150 |  |  |
| 350 mg Q4W | **8.30 (0.52, 16.08)** | 2.09 | 0.037 | 1 | 32 | 31 | NA | NA | NA | NA |
| 280 mg Q4W | 7.40 (-0.38, 15.18) | 1.86 | 0.062 | 1 | 32 | 32 | NA | NA | NA | NA |
| 140 mg Q2W | **4.10 (0.15, 8.05)** | 2.03 | 0.042 | 1 | 51 | 103 | NA | NA | NA | NA |
| % change in ApoA1 at mean of weeks 10 and 12 (EVO vs. EZE) | | | | | | | | | | |
| 420 mg Q4W | 2.70 (-1.15, 6.55) | 1.37 | 0.169 | 1 | 51 | 102 | NA | NA | NA | NA |
| 140 mg Q2W | **5.50 (1.75, 9.25)** | 2.87 | 0.004 | 1 | 51 | 103 | NA | NA | NA | NA |

CI = confidence interval; ApoA1 = apolipoprotein A1; EVO = evolocumab; EZE = ezetimibe; NA = not applicable; PBO = placebo.


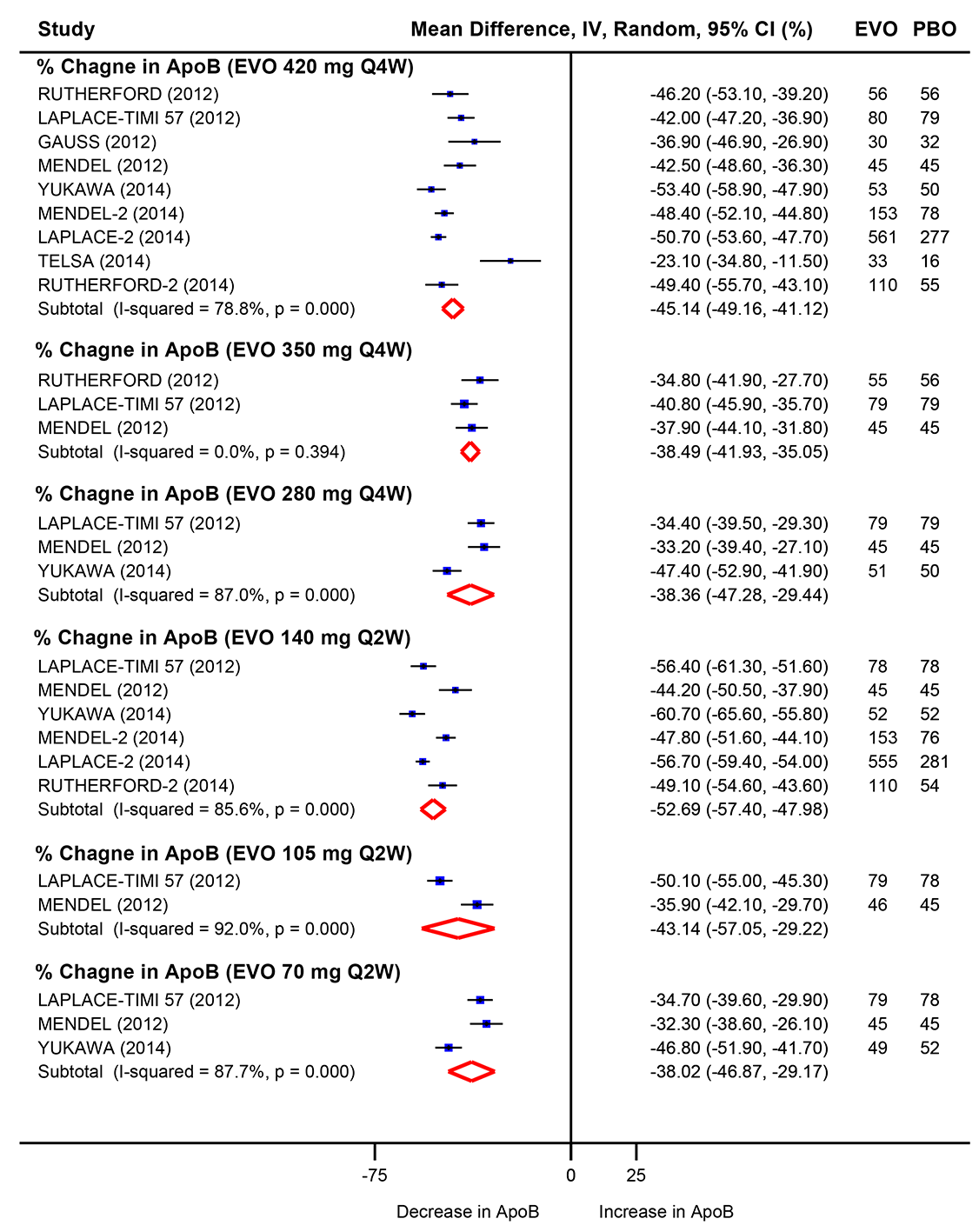


**Figure S7. Forest plot demonstrating changes in apolipoprotein B (ApoB) stratified by dosages following evolocumab treatments versus placebo at 12 weeks follow-up.** CI = confidence interval; ApoB = apolipoprotein B; EVO = evolocumab; PBO = placebo. Other abbreviations as in Table S1.

**Table S12. Percent change of apolipoprotein B (ApoB) at week 12 and at mean of weeks 10 and 12 after evolocumab treatment versus placebo or ezetimibe.**

| **EVO dose** | **Mean Difference (95% CI)** | **Test for overall effect** | | **No. of studies** | **No. of individuals** | | **Heterogeneity** | | **Publication bias** | |
| --- | --- | --- | --- | --- | --- | --- | --- | --- | --- | --- |
|  |  | ***Z*** | ***P* value** |  | **PBO/EZE** | **EVO** | ***I^2^*** | ***P* value** | ***P*_Begg** | ***P*_Egger** |
| % change in ApoB at 12 weeks follow-up (EVO vs. PBO) | | | | | | | | | | |
| 420 mg Q4W | **-45.14 (-49.16, -41.12)** | 22.00 | 0.000 | 9 | 688 | 1121 | 78.8% | 0.000 | 0.076 | 0.027 |
| 350 mg Q4W | **-38.49 (-41.93, -35.05)** | 21.96 | 0.000 | 3 | 180 | 179 | 0.0% | 0.394 | 0.296 | 0.012 |
| 280 mg Q4W | **-38.36 (-47.28, -29.44)** | 8.43 | 0.000 | 3 | 174 | 175 | 87.0% | 0.000 | 1.000 | 0.952 |
| 140 mg Q2W | **-52.69 (-57.40, -47.98)** | 21.91 | 0.000 | 6 | 586 | 993 | 85.6% | 0.000 | 0.707 | 0.450 |
| 105 mg Q2W | **-43.14 (-57.05, -29.22)** | 6.08 | 0.000 | 2 | 123 | 125 | 92.0% | 0.000 | 1.000 | NA |
| 70 mg Q2W | **-38.02 (-46.87, -29.17)** | 8.42 | 0.000 | 3 | 175 | 173 | 87.7% | 0.000 | 1.000 | 0.778 |
| % change in ApoB at mean of weeks 10 and 12 (EVO vs. PBO) | | | | | | | | | | |
| 420 mg Q4W | **-50.33 (-56.15, -44.52)** | 16.97 | 0.000 | 5 | 476 | 910 | 89.5% | 0.000 | 0.221 | 0.145 |
| 280 mg Q4W | **-53.20 (-57.70, -48.70)** | 23.17 | 0.000 | 1 | 50 | 51 | NA | NA | NA | NA |
| 140 mg Q2W | **-53.02 (-58.41, -47.63)** | 19.29 | 0.000 | 4 | 463 | 870 | 88.4% | 0.000 | 1.000 | 0.699 |
| 70 mg Q2W | **-46.40 (-50.80, -42.00)** | 20.67 | 0.000 | 1 | 52 | 49 | NA | NA | NA | NA |
| % change in ApoB at 12 weeks follow-up (EVO vs. EZE) | | | | | | | | | | |
| 420 mg Q4W | **-33.22 (-35.64, -30.79)** | 26.88 | 0.000 | 4 | 269 | 507 | 0.0% | 0.729 | 1.000 | 0.650 |
| 350 mg Q4W | **-22.10 (-31.50, -12.70)** | 4.61 | 0.000 | 1 | 32 | 31 | NA | NA | NA | NA |
| 280 mg Q4W | **-21.40 (-30.80, -12.00)** | 4.46 | 0.000 | 1 | 32 | 32 | NA | NA | NA | NA |
| 140 mg Q2W | **-34.38 (-36.92, -31.83)** | 26.45 | 0.000 | 3 | 240 | 475 | 0.0% | 0.627 | 1.000 | 0.996 |
| % change in ApoB at mean of weeks 10 and 12 (EVO vs. EZE) | | | | | | | | | | |
| 420 mg Q4W | **-36.34 (-39.57, -33.10)** | 22.01 | 0.000 | 3 | 237 | 475 | 47.8% | 0.147 | 1.000 | 0.674 |
| 140 mg Q2W | **-33.47 (-35.83, -31.10)** | 27.76 | 0.000 | 3 | 240 | 475 | 0.0% | 0.804 | 1.000 | 0.626 |

CI = confidence interval; ApoB = apolipoprotein B; EVO = evolocumab; EZE = ezetimibe; NA = not applicable; PBO = placebo.


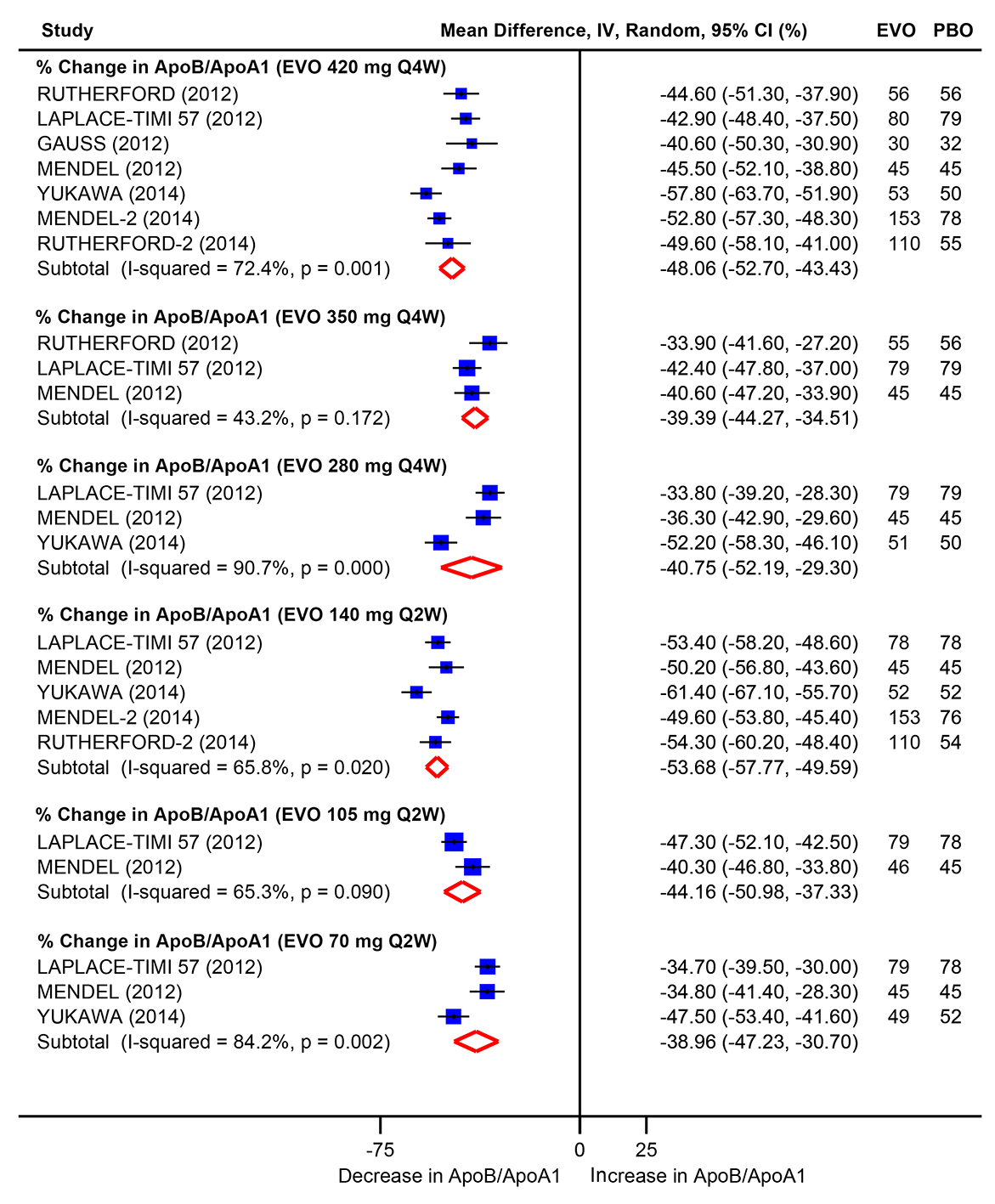


**Figure S8. Forest plot demonstrating changes in ApoB/ApoA1 ratio stratified by dosages following evolocumab treatments versus placebo at 12 weeks follow-up.** CI = confidence interval; ApoB/ApoA1 = apolipoprotein B/apolipoprotein A1 ratio; EVO = evolocumab; PBO = placebo. Other abbreviations as in Table S1.

**Table S13. Percent change of ApoB/ApoA1 ratio at week 12 and at mean of weeks 10 and 12 after evolocumab treatment versus placebo or ezetimibe**.

| **EVO dose** | **Mean Difference (95% CI)** | **Test for overall effect** | | **No. of studies** | **No. of individuals** | | **Heterogeneity** | | **Publication bias** | |
| --- | --- | --- | --- | --- | --- | --- | --- | --- | --- | --- |
|  |  | ***Z*** | ***P* value** |  | **PBO/EZE** | **EVO** | ***I^2^*** | ***P* value** | ***P*_Begg** | ***P*_Egger** |
| % change in ApoB/ApoA1 at 12 weeks follow-up (EVO vs. PBO) | | | | | | | | | | |
| 420 mg Q4W | **-48.06 (-52.70, -43.43)** | 20.32 | 0.000 | 7 | 395 | 527 | 72.4% | 0.001 | 0.649 | 0.351 |
| 350 mg Q4W | **-39.39 (-44.27, -34.51)** | 15.82 | 0.000 | 3 | 180 | 179 | 43.2% | 0.172 | 0.540 | 0.496 |
| 280 mg Q4W | **-40.75 (-52.19, -29.30)** | 6.98 | 0.000 | 3 | 174 | 175 | 90.7% | 0.000 | 1.000 | 0.856 |
| 140 mg Q2W | **-53.68 (-57.77, -49.59)** | 25.74 | 0.000 | 5 | 305 | 438 | 65.8% | 0.020 | 0.806 | 0.500 |
| 105 mg Q2W | **-44.16 (-50.98, -37.33)** | 12.68 | 0.000 | 2 | 123 | 125 | 65.3% | 0.090 | 1.000 | NA |
| 70 mg Q2W | **-38.96 (-47.23, -30.70)** | 9.24 | 0.000 | 3 | 175 | 173 | 84.2% | 0.002 | 1.000 | 0.772 |
| % change in ApoB/ApoA1 at mean of weeks 10 and 12 (EVO vs. PBO) | | | | | | | | | | |
| 420 mg Q4W | **-55.22 (-58.53, -51.90)** | 32.67 | 0.000 | 2 | 133 | 263 | 0.0% | 0.864 | 1.000 | NA |
| 140 mg Q2W | **-50.80 (-54.76, -46.84)** | 25.15 | 0.000 | 2 | 130 | 263 | 33.3% | 0.221 | 1.000 | NA |
| % change in ApoB/ApoA1 at 12 weeks follow-up (EVO vs. EZE) | | | | | | | | | | |
| 420 mg Q4W | **-34.03 (-37.33, -30.74)** | 20.26 | 0.000 | 3 | 160 | 287 | 0.0% | 1.000 | 1.000 | 0.938 |
| 350 mg Q4W | **-26.80 (-35.80, -17.80)** | 5.84 | 0.000 | 1 | 32 | 31 | NA | NA | NA | NA |
| 280 mg Q4W | **-25.10 (-34.00, -16.20)** | 5.53 | 0.000 | 1 | 32 | 32 | NA | NA | NA | NA |
| 140 mg Q2W | **-35.33 (-38.68, -31.98)** | 20.67 | 0.000 | 2 | 128 | 256 | 0.0% | 0.714 | 1.000 | NA |
| % change in ApoB/ApoA1 at mean of weeks 10 and 12 (EVO vs. EZE) | | | | | | | | | | |
| 420 mg Q4W | **-36.52 (-39.83, -33.22)** | 21.67 | 0.000 | 2 | 128 | 255 | 0.0% | 0.954 | 1.000 | NA |
| 140 mg Q2W | **-34.78 (-37.89, -31.67)** | 21.91 | 0.000 | 2 | 128 | 256 | 0.0% | 0.951 | 1.000 | NA |

CI = confidence interval; ApoB/ApoA1 = apolipoprotein B/apolipoprotein A1 ratio; EVO = evolocumab; EZE = ezetimibe; NA = not applicable; PBO = placebo.


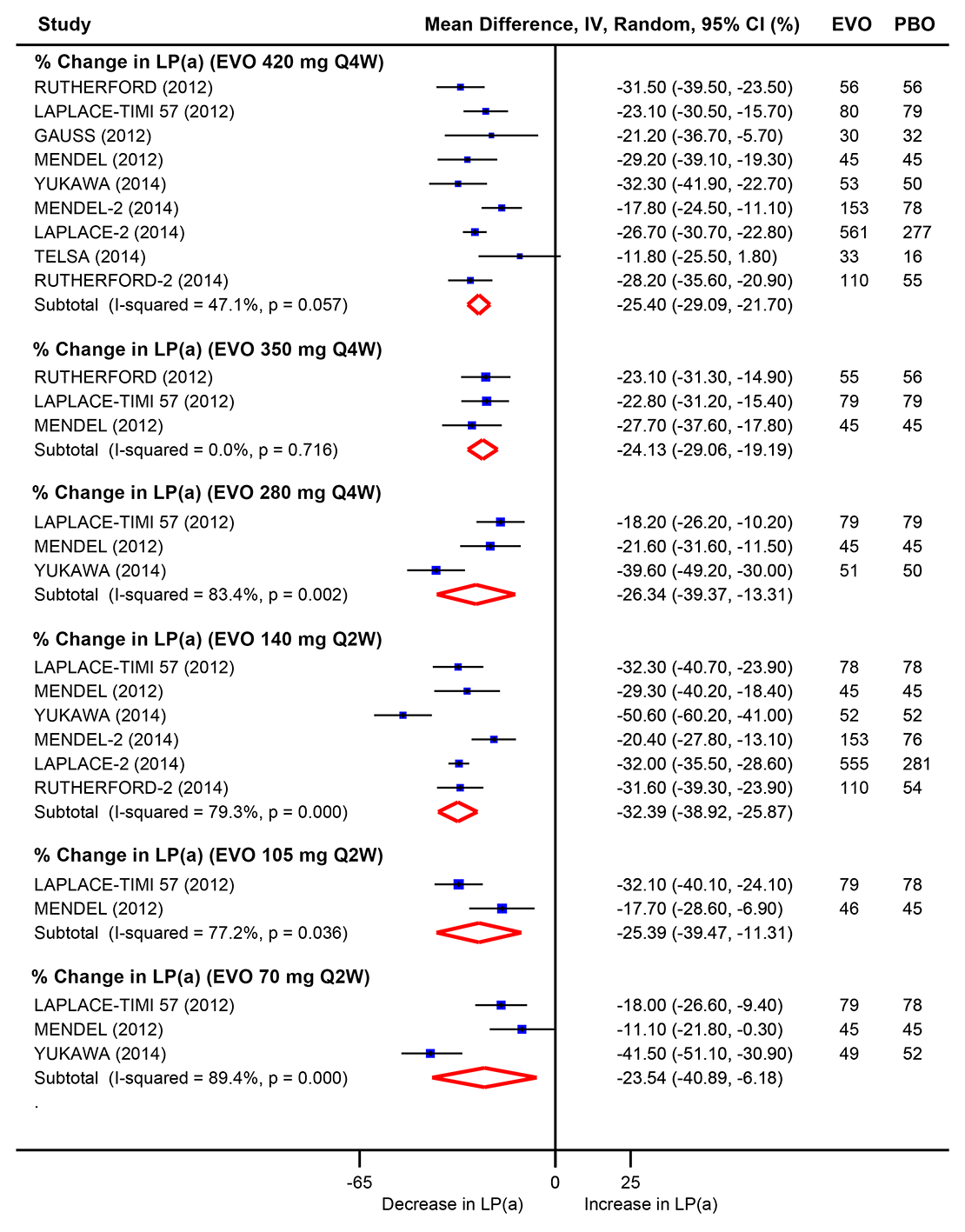


**Figure S9. Forest plot demonstrating changes in lipoprotein(a) (Lp(a)) stratified by dosages following evolocumab treatments versus placebo at 12 weeks follow-up.** CI = confidence interval; EVO = evolocumab; Lp(a) = lipoprotein(a); PBO = placebo. Other abbreviations as in Table S1.

**Table S14. Percent change of lipoprotein(a) (Lp(a)) at week 12 and at mean of weeks 10 and 12 after evolocumab treatment versus placebo or ezetimibe**.

| **EVO dose** | **Mean Difference (95% CI)** | **Test for overall effect** | | **No. of studies** | **No. of individuals** | | **Heterogeneity** | | **Publication bias** | |
| --- | --- | --- | --- | --- | --- | --- | --- | --- | --- | --- |
|  |  | ***Z*** | ***P* value** |  | **PBO/EZE** | **EVO** | ***I^2^*** | ***P* value** | ***P*_Begg** | ***P*_Egger** |
| % change in Lp(a) at 12 weeks follow-up (EVO vs. PBO) | | | | | | | | | | |
| 420 mg Q4W | **-25.40 (-29.09, -21.70)** | 13.47 | 0.000 | 9 | 688 | 1121 | 47.1% | 0.057 | 1.000 | 0.626 |
| 350 mg Q4W | **-24.13 (-29.06, -19.19)** | 9.59 | 0.000 | 3 | 180 | 179 | 0.0% | 0.716 | 0.296 | 0.188 |
| 280 mg Q4W | **-26.34 (-39.37, -13.31)** | 3.96 | 0.000 | 3 | 174 | 175 | 83.4% | 0.002 | 1.000 | 0.648 |
| 140 mg Q2W | **-32.39 (-38.92, -25.87)** | 9.73 | 0.000 | 6 | 586 | 993 | 79.3% | 0.000 | 1.000 | 0.819 |
| 105 mg Q2W | **-25.39 (-39.47, -11.31)** | 3.53 | 0.000 | 2 | 123 | 125 | 77.2% | 0.036 | 1.000 | NA |
| 70 mg Q2W | **-23.54 (-40.89, -6.18)** | 2.66 | 0.008 | 3 | 175 | 173 | 89.4% | 0.000 | 1.000 | 0.818 |
| % change in Lp(a) at mean of weeks 10 and 12 (EVO vs. PBO) | | | | | | | | | | |
| 420 mg Q4W | **-23.01 (-29.37, -16.65)** | 7.09 | 0.000 | 4 | 426 | 857 | 80.6% | 0.001 | 1.000 | 0.848 |
| 140 mg Q2W | **-26.93 (-34.11, -19.76)** | 7.36 | 0.000 | 3 | 411 | 818 | 79.8% | 0.007 | 1.000 | 0.638 |
| % change in Lp(a) at 12 weeks follow-up (EVO vs. EZE) | | | | | | | | | | |
| 420 mg Q4W | **-23.19 (-31.96, -14.43)** | 5.19 | 0.000 | 4 | 269 | 507 | 72.4% | 0.012 | 0.734 | 0.352 |
| 350 mg Q4W | -12.40 (-24.90, 0.10) | 1.94 | 0.052 | 1 | 32 | 31 | NA | NA | NA | NA |
| 280 mg Q4W | **-18.00 (-30.60, -5.40)** | 2.80 | 0.005 | 1 | 32 | 32 | NA | NA | NA | NA |
| 140 mg Q2W | **-26.01 (-32.35, -19.66)** | 8.03 | 0.000 | 3 | 240 | 475 | 59.4% | 0.085 | 1.000 | 0.311 |
| % change in Lp(a) at mean of weeks 10 and 12 (EVO vs. EZE) | | | | | | | | | | |
| 420 mg Q4W | **-25.37 (-35.79, -14.95)** | 4.77 | 0.000 | 3 | 237 | 475 | 86.5% | 0.001 | 1.000 | 0.873 |
| 140 mg Q2W | **-24.15 (-31.20, -17.10)** | 6.71 | 0.000 | 3 | 240 | 475 | 75.1%, | 0.018 | 1.000 | 0.626 |

CI = confidence interval; EVO = evolocumab; EZE = ezetimibe; Lp(a) = lipoprotein(a); NA = not applicable; PBO = placebo.


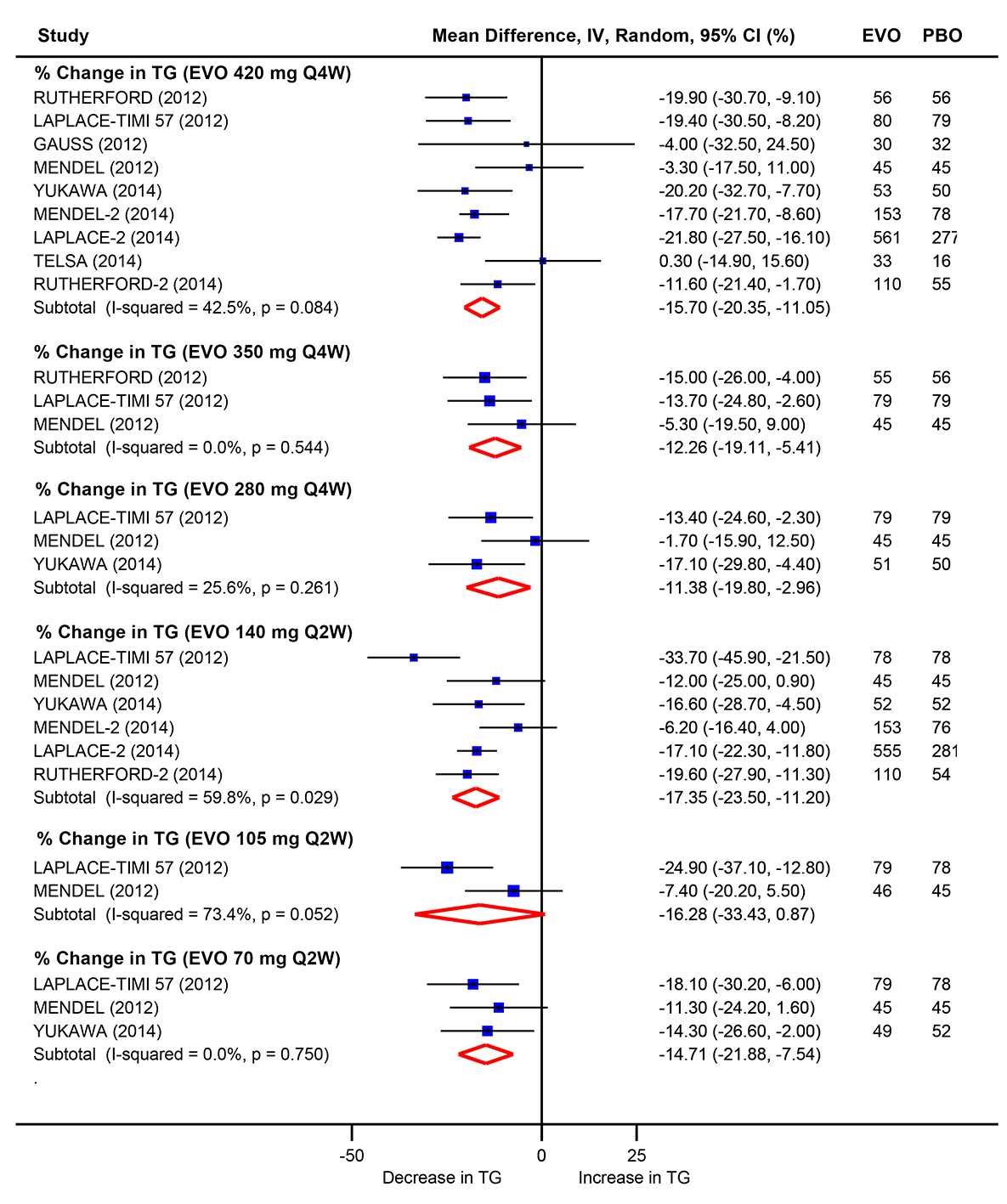


**Figure S10. Forest plot demonstrating changes in triglycerides (TG) stratified by dosages following evolocumab treatments versus placebo at 12 weeks follow-up.** CI = confidence interval; EVO = evolocumab; PBO = placebo; TG = triglycerides. Other abbreviations as in Table S1.

**Table S15. Percent change of triglycerides (TG) at week 12 and at mean of weeks 10 and 12 after evolocumab treatment versus placebo or ezetimibe**.

| **EVO dose** | **Mean Difference (95% CI)** | **Test for overall effect** | | **No. of studies** | **No. of individuals** | | **Heterogeneity** | | **Publication bias** | |
| --- | --- | --- | --- | --- | --- | --- | --- | --- | --- | --- |
|  |  | ***Z*** | ***P* value** |  | **PBO/EZE** | **EVO** | ***I^2^*** | ***P* value** | ***P*_Begg** | ***P*_Egger** |
| % change in TG at 12 weeks follow-up (EVO vs. PBO) | | | | | | | | | | |
| 420 mg Q4W | **-15.70 (-20.35, -11.05)** | 6.62 | 0.000 | 9 | 688 | 1121 | 42.5% | 0.084 | 0.118 | 0.030 |
| 350 mg Q4W | **-12.26 (-19.11, -5.41)** | 3.51 | 0.000 | 3 | 180 | 179 | 0.0% | 0.544 | 0.296 | 0.075 |
| 280 mg Q4W | **-11.38 (-19.80, -2.96)** | 2.65 | 0.008 | 3 | 174 | 175 | 25.6% | 0.261 | 1.000 | 0.537 |
| 140 mg Q2W | **-17.35 (-23.50, -11.20)** | 5.53 | 0.000 | 6 | 586 | 993 | 59.8% | 0.029 | 1.000 | 0.039 |
| 105 mg Q2W | -16.28 (-33.43, 0.87) | 1.86 | 0.063 | 2 | 123 | 125 | 73.4% | 0.052 | 1.000 | NA |
| 70 mg Q2W | **-14.71 (-21.88, -7.54)** | 4.02 | 0.000 | 3 | 175 | 173 | 0.0% | 0.750 | 0.296 | 0.213 |
| % change in TG at mean of weeks 10 and 12 (EVO vs. PBO) | | | | | | | | | | |
| 420 mg Q4W | **-17.88 (-23.29, -12.47)** | 6.47 | 0.000 | 5 | 476 | 910 | 49.2% | 0.096 | 0.221 | 0.116 |
| 280 mg Q4W | **-17.20 (-28.00, -6.40)** | 3.12 | 0.002 | 1 | 50 | 51 | NA | NA | NA | NA |
| 140 mg Q2W | **-15.92 (-22.53, -9.30)** | 4.71 | 0.000 | 4 | 463 | 870 | 71.4% | 0.015 | 0.734 | 0.711 |
| 70 mg Q2W | **-16.50 (-26.50, -6.50)** | 3.23 | 0.001 | 1 | 52 | 49 | NA | NA | NA | NA |
| % change in TG at 12 weeks follow-up (EVO vs. EZE) | | | | | | | | | | |
| 420 mg Q4W | **-9.59 (-15.03, -4.16)** | 3.46 | 0.001 | 4 | 269 | 507 | 0.0% | 0.705 | 0.734 | 0.504 |
| 350 mg Q4W | -13.80 (-34.50, 6.90) | 1.31 | 0.191 | 1 | 32 | 31 | NA | NA | NA | NA |
| 280 mg Q4W | -12.50 (-33.00, 8.00) | 1.20 | 0.232 | 1 | 32 | 32 | NA | NA | NA | NA |
| 140 mg Q2W | -3.70 (-9.12, 1.72) | 1.34 | 0.181 | 3 | 240 | 475 | 13.0% | 0.317 | 0.296 | 0.094 |
| % change in TG at mean of weeks 10 and 12 (EVO vs. EZE) | | | | | | | | | | |
| 420 mg Q4W | **-8.76 (-13.85, -3.68)** | 3.38 | 0.001 | 3 | 237 | 475 | 0.0% | 0.740 | 1.000 | 0.978 |
| 140 mg Q2W | -3.84 (-9.12, 1.44) | 1.43 | 0.154 | 3 | 240 | 475 | 0.0% | 0.591 | 1.000 | 0.936 |

CI = confidence interval; EVO = evolocumab; EZE = ezetimibe; NA = not applicable; PBO = placebo; TG = triglycerides.

**Table S16. Percent change of PCSK9 at week 12 after evolocumab treatment versus placebo.**

| **EVO dose** | **Mean Difference (95% CI)** | **Test for overall effect** | | **No. of studies** | **No. of individuals** | | **Heterogeneity** | | **Publication bias** | |
| --- | --- | --- | --- | --- | --- | --- | --- | --- | --- | --- |
|  |  | ***Z*** | ***P* value** |  | **PBO** | **EZE** | ***I^2^*** | ***P* value** | ***P*_Begg** | ***P*_Egger** |
| % change in PCSK9 at 12 weeks follow-up (EVO vs. PBO) | | | | | | | | | | |
| 420 mg Q4W | **-44.04 (-53.90, -34.17)** | 8.75 | 0.000 | 6 | 540 | 908 | 85.2% | 0.000 | 0.452 | 0.473 |
| 350 mg Q4W | **-46.43 (-51.73, -41.13)** | 17.16 | 0.000 | 2 | 135 | 134 | 0.0% | 0.610 | 1.000 | NA |
| 280 mg Q4W | **-46.30 (-53.25, -39.35)** | 13.06 | 0.000 | 1 | 79 | 79 | NA | NA | NA | NA |
| 140 mg Q2W | **-60.92 (-83.94, -37.89)** | 5.18 | 0.000 | 2 | 132 | 188 | 92.9% | 0.000 | 1.000 | NA |
| 105 mg Q2W | **-65.80 (-73.45, -58.15)** | 16.86 | 0.000 | 1 | 78 | 79 | NA | NA | NA | NA |
| 70 mg Q2W | **-55.40 (-63.10, -47.70)** | 14.10 | 0.000 | 1 | 78 | 79 | NA | NA | NA | NA |

CI = confidence interval; EVO = evolocumab; NA = not applicable; PBO = placebo; PCSK9 = proprotein convertase subtilisin/kexin type 9.


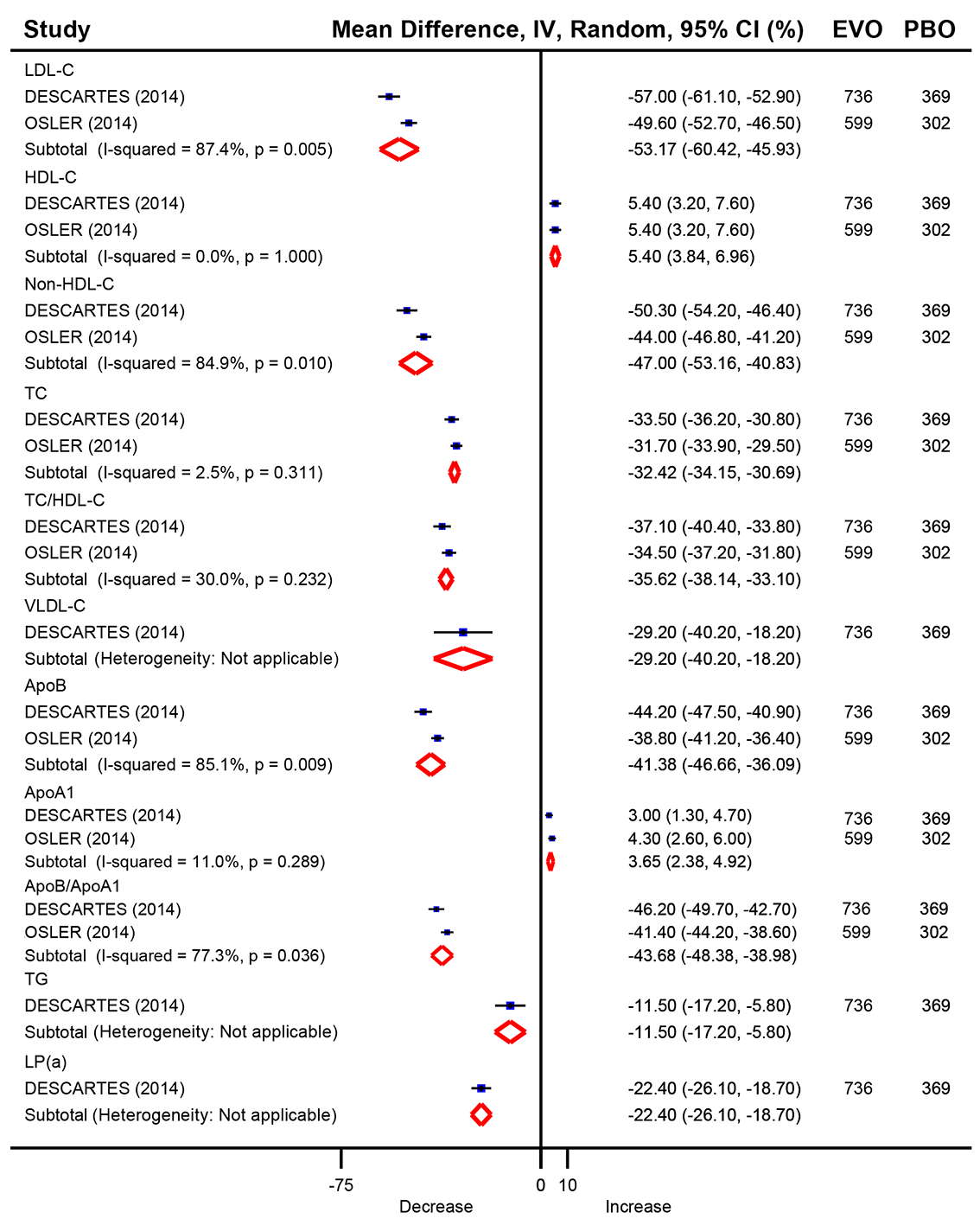


**Figure S11. Forest plot demonstrating changes in lipid profiles following monthly 420 mg evolocumab treatments versus placebo at 52 weeks follow-up.** ApoA1 = apolipoprotein A1; ApoB = apolipoprotein B; ApoB/ApoA1 = ratio of ApoB/ApoA1; EVO = evolocumab; HDL-C = high-density lipoprotein (HDL) cholesterol; Lp(a) = lipoprotein(a); Non-HDL-C = Non-HDL cholesterol; PBO = placebo; TC = total cholesterol; TC/HDL-C = ratio of total cholesterol/HDL cholesterol; TG = triglycerides; VLDL-C = very low-density lipoprotein (VLDL) cholesterol. Other abbreviations as in Table S1.

**Table S17. Percent changes of other endpoints following alirocumab treatment versus placebo or ezetimibe.**

| **Endpoint** | **ALIR dose** | **Mean Difference (95% CI)** | **No. of studies** | **No. of individuals** | |
| --- | --- | --- | --- | --- | --- |
|  |  |  |  | **ALIR** | **PBO/EZE** |
| TC | 50-150 mg Q2W vs. PBO | **-34.48 (-39.37, -29.58)** | 2 | 108 | 46 |
| TC | 75-150 mg Q2W vs. EZE | **-18.70 (-24.70, -12.70)** | 1 | 52 | 51 |
| TC | 150-300 mg Q4W vs. PBO | **-20.14 (-34.44, -5.83)** | 2 | 104 | 46 |
| Non-HDL-C | 50-150 mg Q2W vs. PBO | **-45.78 (-52.98, -38.58)** | 4 | 1870 | 941 |
| Non-HDL-C | 75-150 mg Q2W vs. EZE | **-25.86 (-30.05, -21.68)** | 2 | 178 | 175 |
| Non-HDL-C | 150-300 mg Q4W vs. PBO | **-28.49 (-45.34, -11.64)** | 2 | 104 | 46 |
| ApoB | 50-150 mg Q2W vs. PBO | **-44.78 (-53.45, -36.12)** | 4 | 1870 | 941 |
| ApoB | 75-150 mg Q2W vs. EZE | **-25.29 (-29.21, -21.36)** | 2 | 178 | 175 |
| ApoB | 150-300 mg Q4W vs. PBO | **-25.17 (-41.05, -9.30)** | 2 | 104 | 46 |
| ApoA1 | 50-150 mg Q2W vs. PBO | 8.22 (-2.66, 19.10) | 2 | 46 | 46 |
| ApoA1 | 75-150 mg Q2W vs. EZE | **5.30 (0.85, 9.75)** | 1 | 52 | 51 |
| ApoA1 | 150-300 mg Q4W vs. PBO | **8.50 (3.75, 13.25)** | 1 | 46 | 15 |
| Lp(a) | 50-150 mg Q2W vs. PBO | **-20.27 (-31.44, -9.10)** | 2 | 1762 | 895 |
| Lp(a) | 75-150 mg Q2W vs. EZE | -12.11 (-26.40, 2.17) | 2 | 178 | 175 |
| TG | 75-150 mg Q2W vs. EZE | -1.2 (-12.7, 10.3) | 1 | 52 | 51 |

ALIR = alirocumab; ApoA1 = apolipoprotein A1; ApoB = apolipoprotein B; CI = confidence interval; EVO = evolocumab; EZE = ezetimibe; Lp(a) = lipoprotein(a); Non-HDL-C = Non- high-density lipoprotein (HDL) cholesterol; PBO = placebo; TC = total cholesterol; TG = triglycerides.

**Additional References**

1. Kastelein JJP, Robinson JG, Farnier M, Krempf M, Langslet G, Lorenzato C, Gipe DA, Baccara-Dinet MT: **Efficacy and Safety of Alirocumab in Patients with Heterozygous Familial Hypercholesterolemia not Adequately Controlled with Current Lipid-Lowering Therapy: Design and Rationale of the ODYSSEY FH Studies**. *Cardiovasc Drug Ther* 2014, **28**:281-289.

2. Moriarty PM, Jacobson TA, Bruckert E, Thompson PD, Guyton JR, Baccara-Dinet MT, Gipe D: **Efficacy and safety of alirocumab, a monoclonal antibody to PCSK9, in statin-intolerant patients: Design and rationale of ODYSSEY ALTERNATIVE, a randomized phase 3 trial**. *Journal of Clinical Lipidology* 2014, **8**:554-561.

3. Colhoun HM, Robinson JG, Farnier M, Cariou B, Blom D, Kereiakes DJ, Lorenzato C, Pordy R, Chaudhari U: **Efficacy and safety of alirocumab, a fully human PCSK9 monoclonal antibody, in high cardiovascular risk patients with poorly controlled hypercholesterolemia on maximally tolerated doses of statins: rationale and design of the ODYSSEY COMBO I and II trials**. *BMC Cardiovasc Disord* 2014, **14**:121.

4. Robinson JG, Colhoun HM, Bays HE, Jones PH, Du Y, Hanotin C, Donahue S: **Efficacy and Safety of Alirocumab as Add-on Therapy in High-Cardiovascular-Risk Patients With Hypercholesterolemia Not Adequately Controlled With Atorvastatin (20 or 40 mg) or Rosuvastatin (10 or 20 mg): Design and Rationale of the ODYSSEY OPTIONS Studies**. *Clin Cardiol* 2014, **37**:597-604.
